# *Withania somnifera* extracts induced attenuation of HIV-1: a mechanistic approach to restrict viral infection

**DOI:** 10.1186/s12985-023-02130-y

**Published:** 2023-08-03

**Authors:** Pratiksha Jadaun, R Harshithkumar, Shraddha Y Gaikwad, Chandrabhan Seniya, Swapnil Borse, Ashish A Gawai, Preeti Chavan-Gautam, Girish Tillu, Anupam Mukherjee

**Affiliations:** 1 ICMR-National AIDS Research Institute, Pune, 411026 MH India; 2 School of Biosciences, Engineering and Technology, VIT Bhopal University, Bhopal, 466114 MP India; 3 CCIH-Interdisciplinary School of Health Sciences, AYUSH-Center of Excellence, Savitribai Phule Pune University, Pune, 411007 MH India; 4 Anuradha College of Pharmacy, Buldana, 443201 MH India

**Keywords:** Anti-HIV-1, *Withania somnifera*, Ashwagandha, Ashwagandhanolide, Withanolide, Withacoagin, Withaferin, Molecular docking

## Abstract

**Background:**

Several anti-retroviral drugs are available against Human immunodeficiency virus type-1, but have multiple adverse side effects. Hence, there is an incessant compulsion for effectual anti-retroviral agents with minimal or no intricacy. Traditionally, natural products have been the most successful source for the development of new medications. *Withania somnifera*, also known as Ashwagandha, is the utmost treasured medicinal plant used in Ayurveda, which holds the potential to give adaptogenic, immunomodulatory, and antiviral effects. However, its effect on HIV-1 replication at the cellular level has never been explored. Herein, we focused on the anti-HIV-1 activity and the probable mechanism of action of hydroalcoholic and aqueous extracts of *Withania somnifera* roots and its phytomolecules.

**Methods:**

The cytotoxicity of the extracts was determined through MTT assay, while the in vitro anti-HIV-1 activity was assessed in TZM-bl cells against the HIV-1 strains of X4 and R5 subtypes. Results were confirmed in peripheral blood mononuclear cells, using the HIV-1 p24 antigen assay. Additionally, the mechanism of action was determined through the Time of Addition assay, which was further validated through the series of enzymatic assays, *i.e.* HIV-1 Integrase, Reverse transcriptase, and Protease assays. To explore the role of the identified active metabolites of *Withania somnifera* in antiretroviral activity, molecular docking analyses were performed against these key HIV-1 replication enzymes.

**Results:**

The hydroalcoholic and aqueous extracts of *Withania somnifera* roots were found to be safer at the sub-cytotoxic concentrations and exhibited their ability to inhibit replication of two primary isolates of HIV-1 through cell-associated and cell-free assays, in dose-dependent kinetics. Several active phytomolecules found in *Withania somnifera* successfully established hydrogens bonds in the active binding pocket site residues responsible for the catalytic activity of HIV replication and therefore, signifying their role in the attenuation of HIV-1 infection as implied through the *in silico* molecular docking studies.

**Conclusions:**

Our research identified both the hydroalcoholic and aqueous extracts of *Withania somnifera* roots as potent inhibitors of HIV-1 infection. The *in silico* analyses also indicated the key components of *Withania somnifera* with the highest binding affinity against the HIV-1 Integrase by 12-Deoxywithastramonolide and 27-Hydroxywithanone, HIV-1 Protease by Ashwagandhanolide and Withacoagin, and HIV-1 Reverse transcriptase by Ashwagandhanolide and Withanolide B, thereby showing possible mechanisms of HIV-1 extenuation. Overall, this study classified the role of *Withania somnifera* extracts and their active compounds as potential agents against HIV-1 infection.

**Supplementary Information:**

The online version contains supplementary material available at 10.1186/s12985-023-02130-y.

## Background

Since the detection of earlier cases in 1981 (Centre for Disease Control and Prevention, USA), HIV/AIDS has been considered one of the most disturbing public health problems for more than three decades. Even though targeted antiretroviral therapy has increased the life span, an absolute cure is still out of reach [1]. In 2021, there were 1.5 million new infections of HIV worldwide, adding together up to 38.4 million people living with HIV [2]. Therefore, effective management for controlling the spread of HIV infection is considered the topmost priority.

The existing antiretroviral therapies are showing promising results, but not enough for the complete eradication of HIV/AIDS [3, 4]. Although ARTs have reduced the viral load, transmission, and morbidity/mortality ratio to a great extent, their mechanism of action showed deleterious effects related to mtDNA, impaired glucose metabolism, hepatotoxicity, and multidrug-resistant strains after a prolonged period of infection [5–9]. In the absence of best treatment options for eradicating HIV infection, there is an urgent need for more focused and novel approaches to develop new antiretrovirals or rediscover the existing therapeutic agents. Natural products continue to be the main sources of new therapeutic agents for the management of infectious diseases, and their study has been considered one of the unbeaten strategies for providing a breakthrough in the field of alternative medicines [10–13]. Hence, pharmacologists are continually drawing attention towards biological studies on plant extracts and isolated phytochemicals due to their growing therapeutic values [14–16].

Ayurveda is an ancient Indian traditional system of medicine. Ayurveda-based Rasayana (meaning: rejuvenating) botanicals have adaptogenic and regenerative potential [17–20]. Thus, using the concepts of Ayurvedic treatment in HIV management could potentially stabilize the destructive control or cell death mechanisms and modulate the immune system simultaneously. The primary focus of Rasayana botanicals (phytoconstituents) is to strengthen the host immune system to fight against pathogens. *Withania somnifera* (L.) Dunal (WS) or Ashwagandha, also known as Indian ginseng or Winter cherry, is one of the most treasured medicinal plants used in Ayurveda for more than 3000 years. Being a member of the Solanaceae family, Ashwagandha is an Ayurvedic herb known worldwide for its numerous beneficial health activities since ancient times. This medicinal plant provides benefits against many human illnesses such as epilepsy, depression, arthritis, diabetes and cancer, and palliative effects such as analgesic, rejuvenating, regenerating, and growth-promoting effects [6, 21–27]. Several clinical trials with the different parts of this medicinal herb have demonstrated pure safety and maximum efficacy in patients suffering from these diseases. It has been used for all human age groups and has not yet been linked to any detrimental consequences [28–30]. In our previous study, the phytochemical profile and composition of *Withania somnifera* roots have been reported in detail [31]. Although Ashwagandha is most frequently used in Indian Ayurvedic medicine, its bioactive components’ potential and the mechanistic aspects of its effects are yet to be discovered. Notably, in this study, we report the antiretroviral activity of *Withania somnifera* root extracts as well as their mode of action against HIV-1 infection. We also identified the active metabolites of the plant that might be proven as the essential negative regulators of HIV-1 replication through *in silico* molecular docking studies.

## Methods

### Plant material and extraction

The *Withania somnifera* root powder was a generous gift from Pharmanza Herbal Pvt. Ltd. The standardization of hydroalcoholic (HA) and aqueous extraction (AQ) was carried out for the WS root powder as described earlier by our research group [31]. The WSHA and WSAQ extracts of *Withania somnifera* were prepared as 10% and 12% of concentrations, respectively, and stored at 4°C for further studies.

### Cells and viruses

TZM-bl cell line, a recombinant HeLa cell containing luciferase genes in the HIV-1 LTR promoter region, was obtained from the National Institutes of Health: AIDS Research and Reference Reagent Program (NIH: ARRRP), and maintained in DMEM (Gibco, MA, USA) containing 10% FBS (Moregate, Bulimba, QLD, Australia) and supplemented with HEPES (Gibco, Waltham, MA, USA), antibiotics (Sigma-Aldrich, St. Louis, MO, USA) at 37°C humidified CO_2_ (5%) incubator. These cells express a high level of CD_4_ receptor and co-receptors like CXCR4 (X4) and CCR5 (R5). At least 80% of confluent cells were used for screening of anti-HIV-1 activity of the phytoextracts.

Peripheral blood mononuclear cells (PBMCs) were isolated from the blood of healthy individuals using density gradient centrifugation with Histopaque (Sigma-Aldrich, St. Louis, MO, USA). The isolated PBMCs were activated using PHA-P (5 µg/mL) (Sigma-Aldrich, St. Louis, MO, USA) in a complete growth medium composed of RPMI 1640 (Gibco, MA, USA) containing 10% FBS and IL-2 (5 U/mL). The HIV-1 stock was developed and the validation of anti-HIV-1 activity was addressed utilizing the activated PBMCs.

HIV-1_VB028_ (R5, Subtype C), an Indian primary isolate, was cultured at the Division of Virology, ICMR-National AIDS Research Institute, Pune, while the other primary isolate HIV-1_UG070_ (X4, Subtype D) was obtained from the NIH: ARRRP. The HIV-1 p24 antigen detection assay (Abcam, Cambridge, UK) was done to quantify the infection efficiency of the viral stocks, prepared using the activated PBMCs. The TZM-bl cell lines were used for titration of the virus stocks, and the Spearman-Kärber technique was used to calculate its TCID_50_ (*i.e.*, 50% of tissue culture infectivity dose) as described previously [32, 33].

### Evaluation of cytotoxicity

The cytotoxicity of the WSHA and WSAQ extracts was determined using TZM-bl cells and activated PBMCs with MTT assay (Sigma-Aldrich, St. Louis, MO, USA) as described previously [12]. In brief, double dilutions of the working stock (1 mg/ml) of each of the extracts were prepared and added to the overnight cultured TZM-bl cells (1×10^4^ cells/well) in 96 well plates. After 48 hrs post-incubation, the cell viability was determined using 20µl (5 mg/ml) of MTT reagent. Similarly, the activated PBMCs were treated with various concentrations of the extracts and incubated for 5 days at 37°C in a humidified 5% CO_2_ incubator. Subsequently, the cell viability was assessed using the MTT reagent as described above. The optical density (OD) value was recorded at 550 nm and 630 nm using a multimode plate reader after the final incubation period. The CC_50_ values were obtained at the concentration where 50% of the cells remain viable in the presence of WSHA and WSAQ extracts using non-linear regression model plotting the concentrations of extracts to X axis and the ratio of live cells to Y axis.

### Cell-associated and cell-free anti-HIV-1 assay

In vitro anti-HIV-1 activity of WSHA and WSAQ extracts was performed based on the range of sub-cytotoxic concentrations as obtained by the MTT assay. In the cell-associated assay, the TZM-bl cells (1×10^4^ cells/well) were first infected with pre-titrated virus stocks of HIV-1_VB028_ and HIV-1_UG070_ for 2 hrs before exposing to the serial dilutions of extracts ranging from 0.016–0.250 mg/ml for WSHA and 0.125–2.000 mg/ml for WSAQ. After 48 hrs post-incubation, the luciferase activity was measured using Britelite plus reagent (Perkin Elmer, Waltham, MA, USA). The EC_50_ value (effective concentration showing 50% virus inhibition) was calculated and compared with the standard drug Azidothymidine (AZT). While the cell-associated assay was conducted on the pre-infected TZM-bl cells, the cell-free anti-HIV-1 assay was done with the viral stocks (HIV-1_UG070_ and HIV-1_VB028_) pre-treated with the serial dilutions of WSHA and WSAQ phytoextracts for 1 hr, before adding on the TZM-bl cells (1×10^4^ cells/well). At 48 hpi, the relative luciferase activity was measured, and the EC_50_ value was determined and compared with the standard drug Dextran Sulphate (DS). All the experiments were carried out independently at least three times to cross-verify the obtained results.

### Confirmatory assays using activated PBMC

For confirmation of the anti-HIV-1 activity of phytoextracts, the PBMCs were infected with the pre-titrated HIV-1_VB028_ (40 TCID_50_) strains and seeded in 96 well plates (0.2×10^4^ cells/well), followed by the addition of sub-toxic concentrations of WSHA and WSAQ. At 5 days post-incubation, the release of infectious virus was examined by HIV-1 p24 detection ELISA kit following the manufacturer’s instructions (Abcam, Cambridge, UK), and the absorbance was measured at a wavelength of 450 nm using an ELISA plate reader (BioRad, Hercules, CA, USA). The 50% inhibition concentrations or the EC_50_ values were calculated using the kit’s positive control and results were compared with the standard anti-HIV-1 drug AZT.

While in the cell-free assay, the HIV-1_VB028_ viral stock (40 TCID_50_) was treated with the serial dilutions of WSHA (0.016–0.250 mg/ml) and WSAQ (0.031–1.000 mg/ml) extracts and incubated for 1 hr. After adding the pre-treated virus to the activated PBMCs (0.2×10^4^ cells/well), the cells were incubated for 24 hrs. The cells were added to the fresh medium after rinsing with PBS on the next day followed by incubation for 5 days at 37°C in a 5% CO_2_ humidified chamber. The virus supernatant was examined for the presence of p24 antigen as previously mentioned, and the EC_50_ values were calculated accordingly. DS was used as a positive control for the cell-free confirmatory assay.

The non-infected cell control (mock) and the untreated HIV-1 infected cells (Virus control or VC) were included in the anti-HIV-1 assays. The preinstalled software of the Perkin Elmer multimode Luminometer system calculated the percentage inhibition of HIV-1 infection of the extracts along with the EC_50_ values using the formula given below:

$$Percentage\,inhibition = \left[ {1 - \frac{{(Avg\,RL{U_{Test}} - Avg\,RL{U_{Mock}})}}{{(Avg\,RL{U_{VC}} - Avg\,RL{U_{Mock}})}}} \right] \times 100$$

### Time-of-addition assay (TOA)

The TOA assay was carried out as previously described with some modifications [34]. TZM-bl cells (1×10^4^ cells/well) were seeded on a 96-well plate. After overnight incubation, the cells were infected with HIV-1_VB028_ along with 25 µg/mL DEAE-dextran (Sigma-Aldrich, St. Louis, MO, USA). Well-known antiretrovirals like Azidothymidine (AZT: A Nucleotide Reverse transcriptase Inhibitors or NRTI − 0.49 µM), Raltegravir (RAL: Integrase inhibitor − 0.48 µM), and Ritonavir (RTV: Protease inhibitor – 10 µM), along with the WSHA (0.029 mg/ml) and WSAQ (0.378 mg/ml) extracts were either added to the wells concurrently (0 hpi) or at different hours of post-infection as indicated (0–24 hpi). The antiretrovirals and extracts were used based on their respective EC_50_ concentrations. At 48 hpi, the luciferase activity was measured using Britelite plus reagent (Perkin Elmer, Waltham, MA, USA), and the means and SD of the experiments were calculated after performing the experiment in triplicate.

### HIV-1 Integrase (INT) inhibition assay

Commercially available HIV-1 Integrase Assay Kit (XpressBio, Frederick, MD, USA) was used to test the inhibitory effects of the WSHA and WSAQ extracts on HIV-1 INT activity. In a nutshell, a double-stranded HIV-1 LTR U5 donor substrate (DS) oligonucleotide with end-labelled biotin was coated onto the Streptavidin-covered 96-well plates. The DS DNA substrate was loaded with full-length recombinant HIV-1 INT protein. A new double-stranded target substrate (TS) DNA with a 3′-end alteration was added to the enzyme reaction after the addition of WSHA and WSAQ extracts. 1.0% Sodium Azide (provided with the kit PC) and known HIV-1 INT inhibitor, RAL (0.48 µM), were used as a positive control, whereas the Integrase (provided with the kit as NC) was used as a negative control. The HIV-1 IN cleaves the last two bases from the exposed 3′ end of the HIV-1 LTR DS DNA, which is subsequently integrated into the TS DNA through a strand-transfer recombination process. The reaction’s products were identified calorimetrically using an HRP-labeled antibody that was directed against the TS 3′-end alteration, and the absorbance caused by the HRP antibody-TMB peroxidase substrate reaction was measured at 450 nm. The percentage inhibition was calculated following the manufacturer’s instruction manual (XpressBio: EZ-1700 Version 3.0).

### HIV-1 Protease (PR) inhibition activity assay

The WSHA and WSAQ extracts were tested for HIV-1 Protease inhibitory activity using an HIV-1 PR inhibitor screening Fluorometric assay kit (Abcam, Cambridge, UK). Briefly, each sample was incubated with the HIV-1 PR enzyme for 15 min at room temperature. The fluorescent substrate was then added to the wells, and the absorbance (excitation/emission = 330/450 nm) was measured using a plate reader in a kinetic mode for 120 min at 37°C. The kit-supplied Enzyme Control (EC) was used as the negative control, whereas the Inhibitor Control (IC) Pepstatin (1 mM) and known Protease inhibitor RTV (10 µM) were used as the positive controls for measuring the inhibition of HIV-1 Protease activity. DMSO (1%, v/v) was used as the vehicle to normalize the background noise. The percentage inhibition of HIV-1 Protease in presence of WSHA and WSAQ extracts was calculated by the Relative Fluorescence Unit (RFU) of each test sample using the formula given below:

$$Percentage\,inhibition = 100 - \left[ {\frac{{(Avg\,\Delta RL{U_{Test}} - Avg\,\Delta RL{U_{Blank}})}}{{(Avg\,\Delta RL{U_{EC}} - Avg\,\Delta RL{U_{Blank}})}}} \right] \times 100$$

### HIV-1 Reverse transcriptase (RT) inhibition activity assay

The inhibitory action of both WSHA and WSAQ extracts on HIV-1 Reverse transcriptase (RT) was assessed using a commercially available kit (Roche, Penzber, Germany). Briefly, dilutions of the extracts were incubated with the HIV-1 RT in addition to the template nucleotide mixture for 1 hr. Consequently, the mixture was transferred to the microwell plates coated with streptavidin and followed by further incubation of 1 hr to bind with biotin, which is a DIG-labeled template primer complexed to the streptavidin-coated plate. The enzyme HRP conjugate was added to each well followed by another incubation of 1 hr. After the addition of the substrate, absorbance was measured at the wavelength of 405 and 490 nm by using BioRad reader PR4100. AZT, the known inhibitor of HIV-1 RT, was used as a positive control. The resulting signal intensities were directly proportional to the actual HIV-1 RT activities, therefore, to compare the inhibitory activity of the WSHA and WSAQ extracts, percent inhibitions were calculated with respect to the untreated test control following the manufacturer’s instruction manual (Roche: 11468120910 Version 16.0).

### Protein structure selection and preparation

The crystal structures of HIV-1 Integrase (PDB: 1QS4, resolution 2.01 Å), HIV-1 Protease (PDB: 5KR0, resolution 1.8 Å), and HIV-1 Reverse transcriptase (PDB: 3QIP, resolution 2.09 Å) were retrieved from the RCSB protein data bank (http://www.rscb.org). The HIV-1 proteins were processed to obtain a single chain for docking studies by removing water molecules, hetatoms and previously docked or attached ligands using BIOVIA Discovery Studio Visualizer v21.1.0.20298 (https://www.3ds.com/products-services/biovia/products/molecular-modeling-simulation/biovia-discovery-studio/). Further, the process also involves insertion and optimization of polar hydrogen, merging non-polar hydrogen, energy minimization by adding Kollman charges, checking for missing atoms and their repair, optimization of bond lengths, formation of disulphide bonds, capping of protein terminals, and the conversion of selenomethionine to methionine.

### Preparation of ligand for Docking Simulations

The 2D/3D structures confirmations of active phytomolecules of *Withania somnifera*, *viz.* 12-Deoxywithastramonolide (CID:44576309), 27-Hydroxywithanone (CID:21574483), Ashwagandhanolide (CID:16099532), Withacoagin (CID:14236709), Withaferin A (CID:265237), Withanolide A (CID:11294368), Withanolide B (CID:14236711), Withanone (CID:21679027), Withanoside IV (CID:71312551), and Withanoside V (ID:10700345) were downloaded from the PubChem database (https://pubchem.ncbi.nlm.nih.gov/) in SDF format. FDA-approved known HIV-1 Integrase inhibitors, *viz.* Cabotegravir (CID:54713659), Dolutegravir (CID:46216142), and Raltegravir (CID:23668479), HIV-1 Protease inhibitor *viz.* Ritonavir (CID:392622) and HIV-1 Reverse transcriptase inhibitor *viz.* Zidovudine (CID:35370) were also downloaded for comparative analysis. The 2D structures coordinates were converted into 3D using Avogadro for dockings simulations studies against HIV replication proteins [35]. Avogadro tool generates tautomeric, stereochemical, and ionisation modifications, as well as energy minimization and flexible filtering.

### Molecular docking of phytomolecules against HIV-1 targets

The inhibitory potential of phytomolecules of *Withania somnifera* can be identified against HIV-1 proteins using molecular docking simulations. These simulations provide crucial information about molecular interactions between the protein and the ligands of interest and render an opportunity to study their catalytic behaviour. The HIV replication protein PDB and ligand 3D files were fetched in AutoDockTools v4.2. Polar hydrogen(s) were added, non-polar(s) were merged and the Kollman charges were added to the protein, while Gasteiger charges were applied to the ligand molecules for optimization of the ligand energy state. The Torsion tress was checked for choosing torsion settings in AutoDock v4.2 (https://autodock.scripps.edu) [36]. A grid box of 60×60×60 Å with a spacing of 0.5 Å was generated using AutoGrid4 and centered on the active residues in the binding pocket of protein where the ligand of interest can interact under isolated conditions. The Lamarckian Genetic algorithm 4.2 with 10 iterations over 150 populations was used with default docking parameters. *In silico* molecular interactions and conformational analysis were visualized in the PMV tool available in AutoDockTools v1.5.7 and 2D plots were generated in BIOVIA Discovery Studio Visualizer v21.1.0.20298.

### Statistical analysis of in vitro assays

The mean values of minimum two replicates were taken for each experiment. The final results were calculated and represented as the percentage inhibition bar graphs after nullifying the blank and comparing with the respective controls from at least three such independent experiments. The error bars represent the standard deviation of the mean of three assays for each experiment. The p-values were calculated using the ANOVA test using the GraphPad Prism software comparing between the different concentrations of WSHA or WSAQ (Supplementary file 1) and among the different experimental groups (Supplementary file 2). Statistical significances were determined as * p ≤ 0.05, ** p ≤ 0.01, or *** p ≤ 0.001.

## Results

### In vitro cytotoxicity of *Withania somnifera* extracts on TZM-bl cell line and PBMCs

**Fig. 1:**
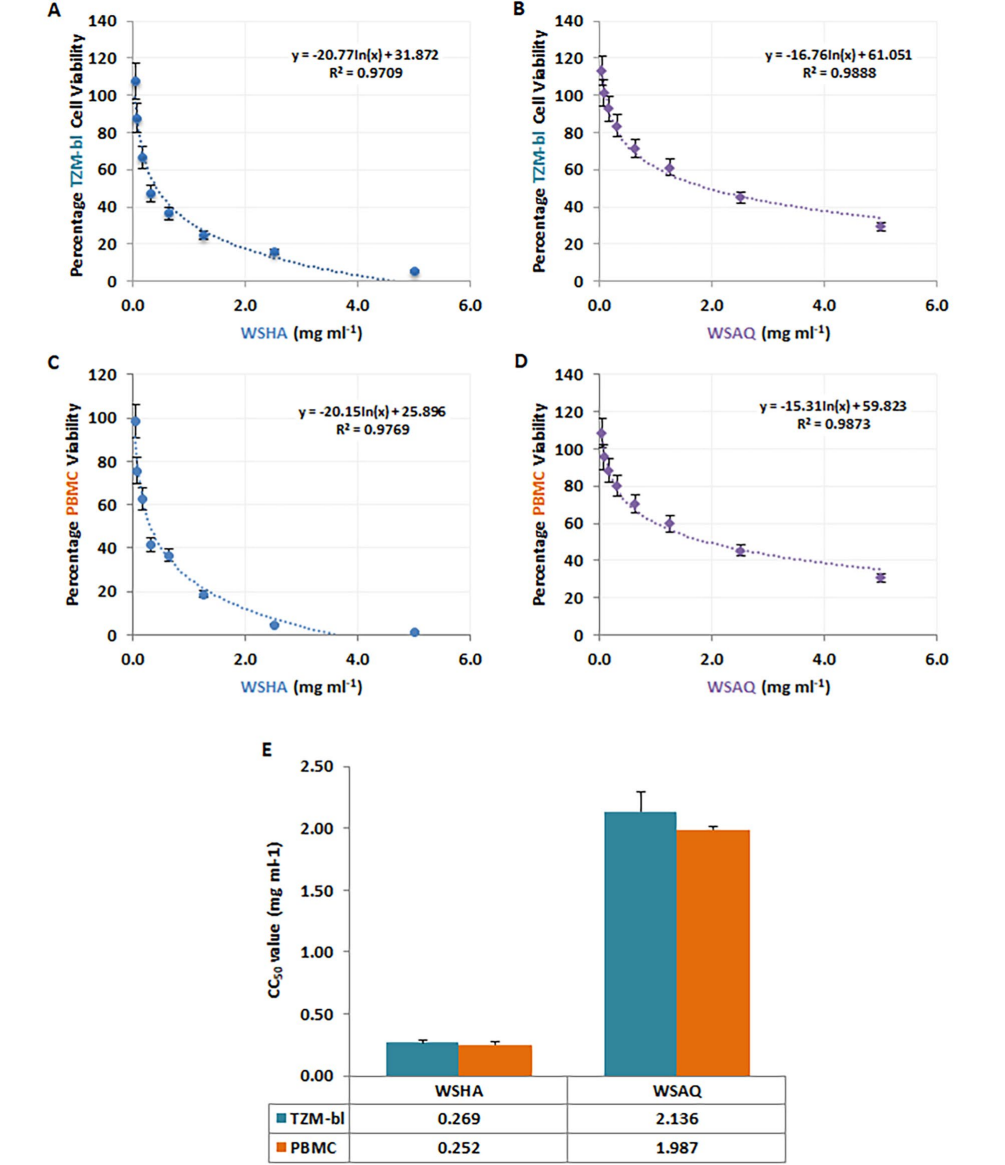
Determination of cytotoxic concentration of *Withania somnifera* hydroalcoholic (WSHA) and aqueous (WSAQ) extracts: The percentage cell viability was examined for (**A**) WSHA and (**B**) WSAQ on TZM-bl cells at 0.039-5.000 mg/ml concentrations. The extract concentration versus percentage viability of PBMCs were also tested for (**C**) WSHA and (**D**) WSAQ extracts in a dose-dependent kinetics. (**E**) Mean CC_50_ values of WSHA and WSAQ were determined and represented as error bar with SD from three independent assays

WSAQ and WSHA extracts were initially examined for their cellular viability on TZM-bl cell lines and PBMCs using MTT quantitative colorimetric assay. Increasing the concentrations directly attributed to the increase in the cytotoxicity for WSHA and WSAQ extracts in the TZM-bl cells (Fig. 1A and B), as well as in the PBMCs (Fig. 1C and D). The CC_50_ values of 0.269 mg/ml and 2.136 mg/ml were determined for WSHA and WSAQ extracts, respectively (Fig. 1E). A similar pattern was observed in PBMCs as well, where CC_50_ values were obtained as 0.252 mg/ml and 1.987 mg/ml, respectively, for the hydroalcoholic and aqueous extracts of *Withania somnifera* (Fig. 1E).

### Anti-HIV-1 activities of *Withania somnifera* extracts

The activity of WSHA and WSAQ against two clades of HIV-1 strains was tested in the TZM-bl cell line and later the same was validated in PBMCs. The concentration below the cytotoxic levels in accordance with CC_50_ values was used for anti-HIV-1 screening. The potential of extracts to inhibit virus replication through cell-associated (CA) as well as cell-free (CF) assays was evaluated subsequently. Azidothymidine (AZT: 0.49 µM) and Dextran sulfate (DS: 15 µg/ml) were used as positive controls in CA and CF assays, respectively.

### Inhibition of HIV-1 infection by the extracts in TZM-bl cells

**Fig. 2:**
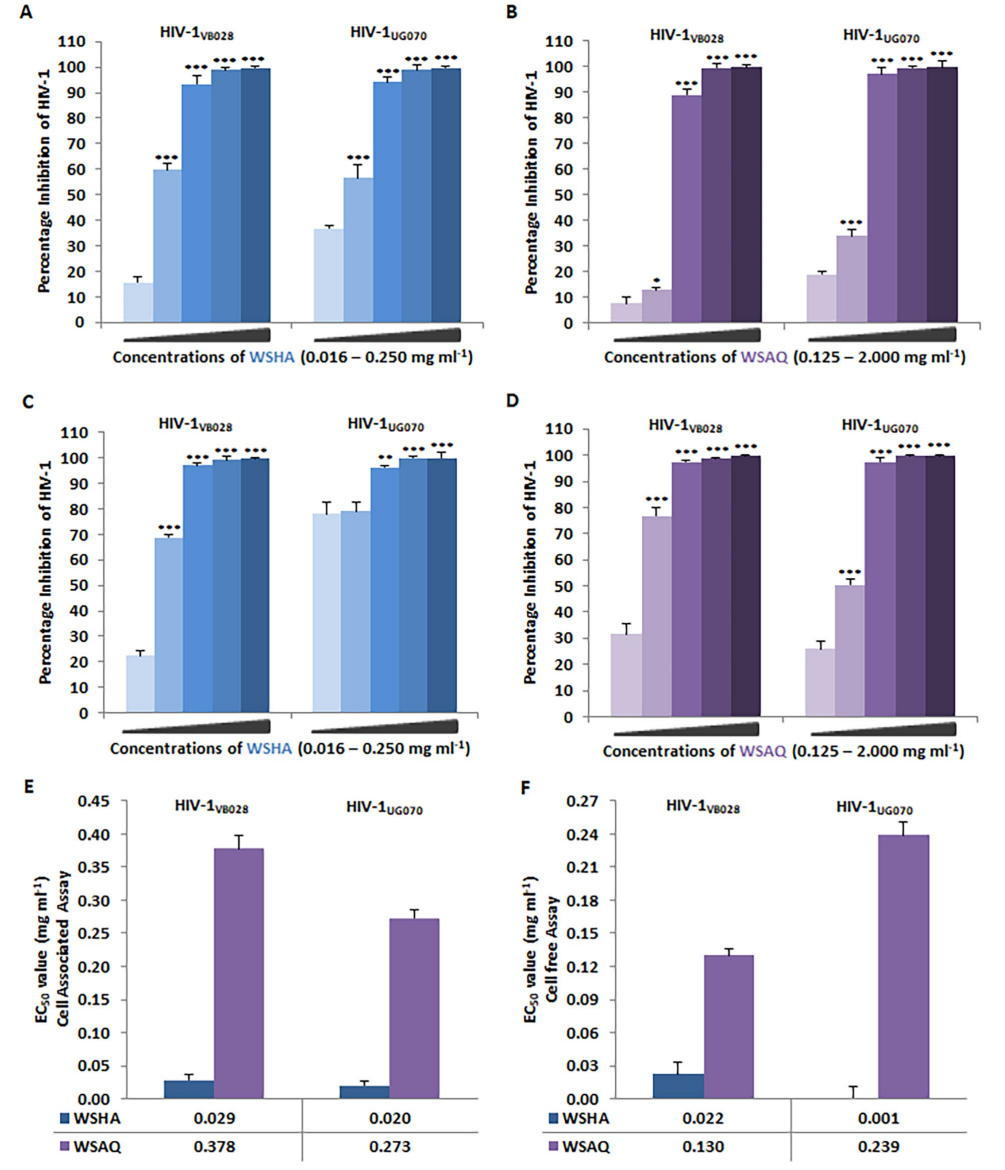
Percentage inhibition of HIV-1 replication by the *Withania somnifera* extracts in TZM-bl cells: Through cell-associate assays (CA) effect of (**A**) WSHA and (**B**) WSAQ on HIV-1_VB028_ (R5, Subtype C) and HIV-1_UG070_ (X4, Subtype D) infected cells. Through cell-free assays (CF) effect of (**C**) WSHA and (**D**) WSAQ on HIV-1_VB028_ and HIV-1_UG070_ infected cells. Comparison between the EC_50_ values of WSHA and WSAQ in two HIV-1 subtypes in (**E**) CA and (**F**) CF assays. The results shown are the means of at least three experimental replicates plus the standard deviations were calculated and represented as the error bar. By combining one-way ANOVA with multiple comparison analysis, statistical significance was observed in all the experiments between the various concentrations in respect to the lowest concentration of WSHA or WSAQ (Supplementary file 1). * p ≤ 0.05, ** p ≤ 0.01, *** p ≤ 0.001

In the cell-associated assays, the pre-infected TZM-bl cells were treated with the extracts of *Withania somnifera*, with a range of multiple concentrations (WSHA: 0.016–0.250 mg/ml, and WSAQ: 0.125–2.000 mg/ml) below the CC_50_ values, showed a clear dose-dependent inhibition of HIV-1 infection for both clades, HIV-1_VB028_ and HIV-1_UG070_ (Fig. 2A and B). While, the cell-free assay, where the virus was introduced to the different concentrations of the extracts before the administration to the cells, showed similar dose-dependent anti-HIV-1 activity in the presence of WSHA (0.016–0.250 mg/ml) and WSAQ (0.125–2.000 mg/ml) (Fig. 2C and D).

The activity of WSHA extract showed excellent inhibition of HIV-1 infection even at the lower dosages, revealing the half maximal effective concentration or EC_50_ values of 0.029 mg/ml and 0.020 mg/ml in CA assays, and 0.022 mg/ml and 0.001 mg/ml in CF assays, for the HIV-1_VB028_ and HIV-1_UG070_, respectively (Fig. 2E and F). Whereas, the EC_50_ values of WSAQ extract were identified as 0.378 mg/ml and 0.273 mg/ml in CA and 0.130 mg/ml and 0.239 mg/ml in CF for the inhibition of HIV-1_VB028_ and HIV-1_UG070_ strains, respectively (Fig. 2E and F).

### Inhibition of HIV-1 infection in peripheral blood mononuclear cells (PBMCs)

**Fig. 3:**
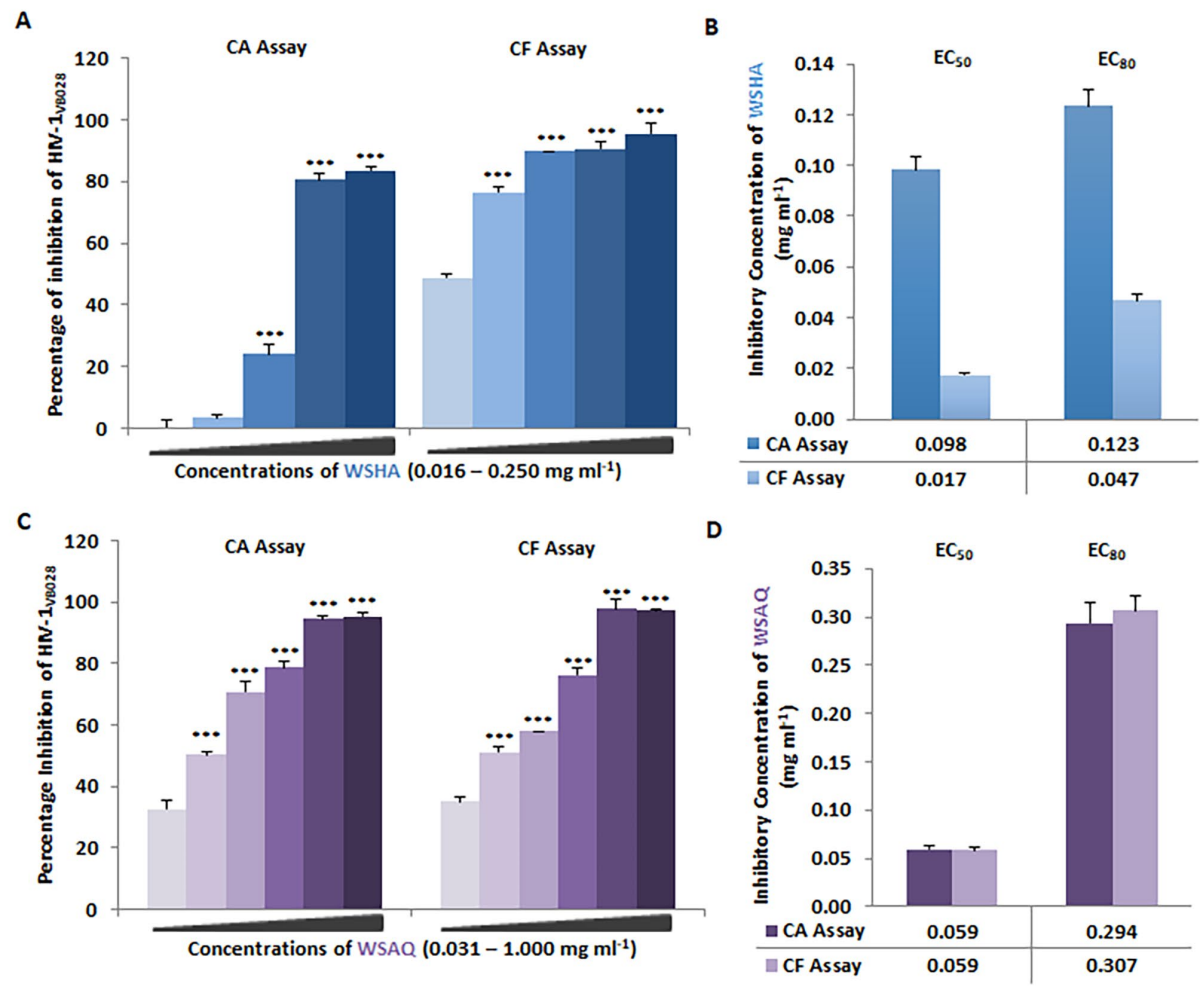
Anti-HIV-1 activity of *Withania somnifera* extracts in HIV-1_VB028_ infected PBMCs: (**A**) Dose-dependent inhibition of HIV-1 infection in cell-associated (CA) and cell-free (CF) assays by *Withania somnifera* hydroalcoholic extract (WSHA). (**B**) Comparative analysis of HIV-1 inhibitory concentrations (EC_50_ & EC_80_) of WSHA through CA and CF assays. (**C**) Dose-dependent inhibition of HIV-1 infection in cell-associated (CA) and cell-free (CF) assays by *Withania somnifera* by WSAQ or the aqueous extract of WS (**D**) The EC_50_ and EC_80_ values of WSAQ are also comparable in both CA and CF assays respectively. The results shown are the means of at least three experimental replicates plus the standard deviations were calculated and represented as the error bar. By combining one-way ANOVA with multiple comparison analysis, statistical significance was determined in all the experiments between the various concentrations in respect to the lowest concentration of WSHA or WSAQ (Supplementary file 1). *** p < 0.001

Consequently, the CA and CF confirmatory assays using the primary isolate HIV-1_VB028_ infected PBMCs, and WSHA extracts at different concentrations (0.016–0.250 mg/ml) showed a dose-dependent inhibition of HIV-1 p24 protein (Fig. 3A). The EC_50_ values for both the CA and CF assays were obtained as 0.098 mg/ml and 0.017 mg/ml, whereas the EC_80_ values as 0.123 mg/ml and 0.047 mg/ml for the WSHA extract in PBMCs (Fig. 3B).

Similarly, the WSAQ extracts also showed a comparable pattern of dose-dependent (0.031–1.000 mg/ml) inhibition of HIV-1 p24 protein in both CA and CF assays (Fig. 3C). However, both the EC_50_ and EC_80_ values of WSAQ in PBMCs were observed to be 0.059 mg/ml and 0.294 mg/ml in the CA assay, and 0.059 mg/ml and 0.307 mg/ml in the CF assay (Fig. 3D).

FDA-approved standard drugs, AZT and DS, showed 100% inhibition of HIV-1 both in the TZM-bl and PBMCs in the in vitro CA or CF assays and confirmatory studies (data not shown).

### Elucidation of the mechanism of action of extracts by TOA assay

**Fig. 4:**
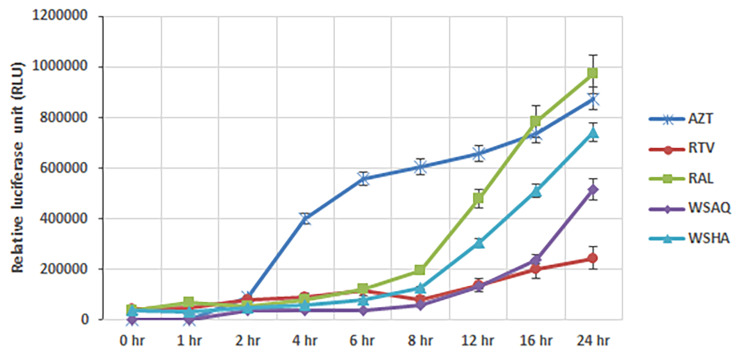
Time-of-addition (TOA) assays were performed with WSHA and WSAQ extracts or the reference inhibitors: AZT (Reverse transcription), RTV (Protease inhibitor) and RAL (Integrase inhibitor). The time point kinetics was assayed as the mean of three independent replicates and the infection levels were normalized to those of untreated controls (= 100%). Symbols indicate the mean values and the error bars as the standard deviation of replicates

The time-of-addition assay (TOA) was conducted for the *Withania somnifera* extracts, WSHA (0.029 mg/ml) and WSAQ (0.378 mg/ml), with known antiretrovirals to determine the targets of these extracts. Final concentrations of drugs and extracts were introduced at various time intervals before and/or after the HIV-1 infection (0, 1, 2, 4, 6, 8, 12, 16, and 24 h). The infection percentage (RLU) was calculated accordingly. It was noted that the extract of WSAQ’s inhibition began to reduce at 16 hpi, but the inhibition of WSHA activity was initiated at 8 hpi. According to the findings, WSHA followed an inhibition pattern similar to the known HIV-1 Integrase inhibitor RAL, whereas, the WSAQ exhibited a pattern similar to the HIV-1 Protease inhibitor RTV (Fig. 4). Hence, it can be predicted that the modus operandi of WSHA and WSAQ might be similar to the known Protease and Integrase inhibitors of HIV-1 inhibition, respectively.

### In vitro inhibition of HIV-1 Integrase by *Withania somnifera* extracts

**Fig. 5:**
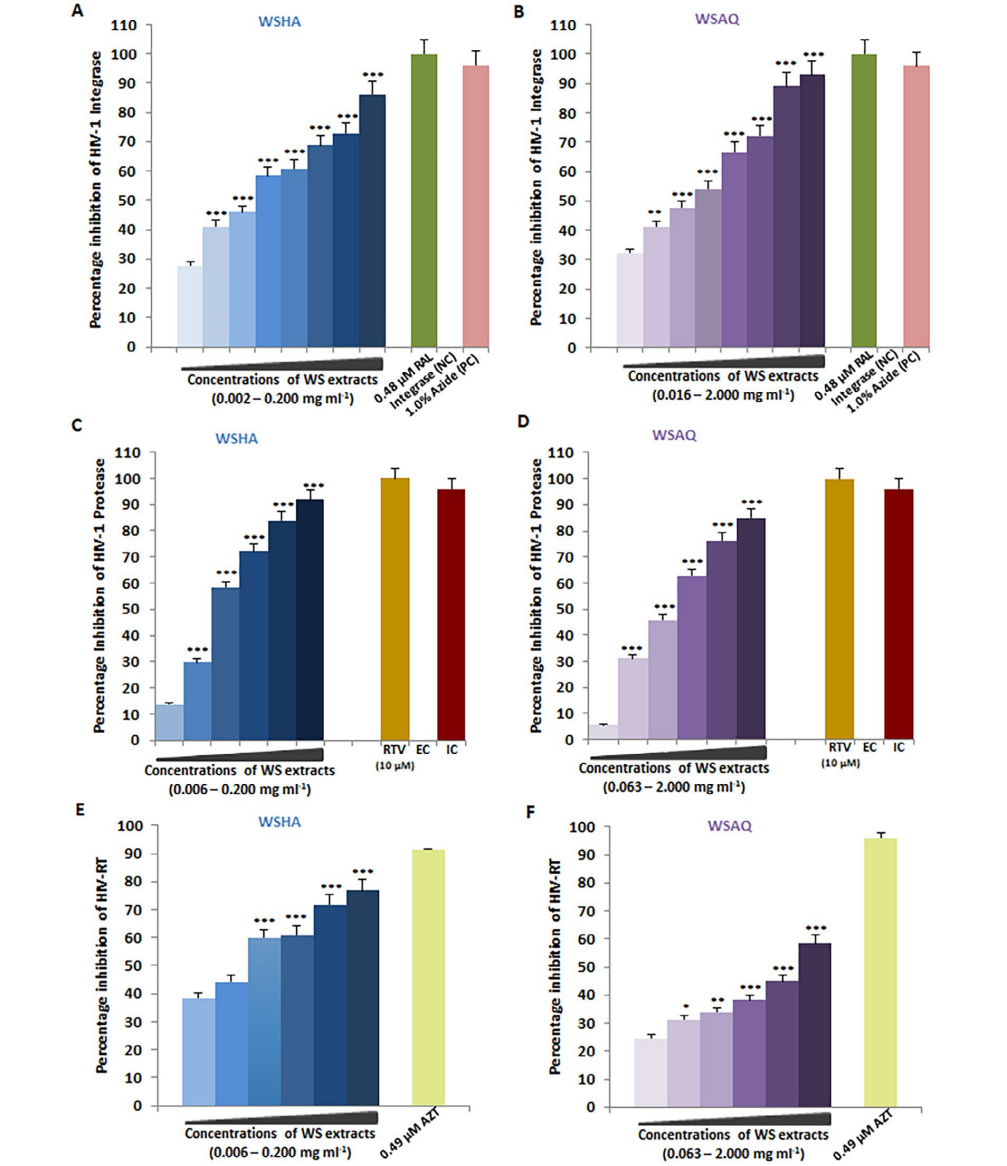
In vitro inhibition of HIV-1 key proteins by *Withania somnifera* hydroalcoholic (WSHA) and aqueous (WSAQ) extracts: (**A** and **B**) Integrase: Percentage inhibition values for the extracts tested against HIV-1 Integrase’s percent activity relative to the kit control Azide (1.0%) and known HIV-1 Integrase inhibitor Raltegravir (0.48 µM). (**C** and **D**) Protease: The percentage of HIV-1 Protease enzyme activity suppression in the presence of extracts was compared to the kit provided Inhibitor Control (IC), Pepstatin (1mM), and the known HIV-1 PR inhibitor RTV (10 µM). To normalize background fluorescence, Enzyme Control (EC) represents the negative control. (**E** and **F**) Reverse transcriptase: Percentage inhibition of HIV-1 RTase enzyme activity in presence of the extracts; as a positive control, AZT (0.49 µM), a known HIV-1 RT inhibitor, was utilized. The results shown as the means of at least three experimental replicates plus the standard deviations were calculated and represented as the error bar. By combining one-way ANOVA with multiple comparison analysis, statistical significance was determined in all the experiments between the various concentrations in respect to the lowest concentration of WSHA or WSAQ (Supplementary file 1). * p ≤ 0.05, ** p ≤ 0.01, *** p < 0.001

To interrogate potential anti-HIV-1 mechanisms of *Withania somnifera* extracts, in vitro HIV-1 Integrase assay was conducted with the different concentrations of WSHA (0.002–0.200 mg/ml) and WSAQ (0.016–2.000 mg/ml). Whereas WSHA showed maximum inhibition of 86.18% at a concentration of 0.200 mg/ml (Fig. 5A), 93.98% inhibition was observed at the highest sub-cytotoxic concentration (2.000 mg/ml) of WSAQ (Fig. 5B). Based on the obtained results of dose-dependent inhibition of HIV-1 Integrase, the EC_50_ values were calculated as 0.010 mg/ml for WSHA and 0.070 mg/ml for WSAQ. The kit supplied positive control, Sodium Azide (1%), exhibited 95.99% inhibition, whereas, Raltegravir (0.48 µM) was taken as an additional inhibitor of HIV-1 Integrase, showed 100.0% inhibition in support of the assay validation.

### In vitro inhibition of HIV-1 Protease by *Withania somnifera* extracts

Furthermore, we examined the activity of WSHA and WSAQ extracts in HIV-1 Protease inhibition through the in vitro kit-based assay with the different concentrations of WSHA (0.006–0.200 mg/ml) and WSAQ (0.063–2.000 mg/ml). The result revealed 91.776% inhibition of HIV-1 Protease activity at 0.200 mg/ml for WSHA extract (Fig. 5C), while 84.840% inhibition for WSAQ at 2.000 mg/ml (Fig. 5D). Based on the obtained results of dose-dependent inhibition of HIV-1 protease, the EC_50_ values were calculated as 0.024 mg/ml and 0.337 mg/ml for WSHA and WSAQ, respectively. The result was compared with the known HIV-1 Protease inhibitor Ritonavir (10 µM) as a positive control with 77.65% inhibition of Protease activity, as well. Further, the assay was validated with the kit-provided Inhibitor control, Pepstatin (1 mM) and Enzyme control (EC).

### In vitro inhibition of HIV-1 Reverse transcriptase by *Withania somnifera* extracts

The *Withania Somnifera* extracts were evaluated for their potential to inhibit HIV Reverse transcriptase as described previously [12]. The WSHA showed 38.46–76.82% inhibition of HIV-1 RT activity in a dose-dependent (0.006–0.200 mg/ml) kinetics assay (Fig. 5E). However, the multiple sub-cytotoxic concentrations of WSAQ extract (0.063–2.000 mg/ml) revealed not-so-promising inhibition of HIV-1 RT activity with a range of 24.63–58.53% only (Fig. 5F). Based on the obtained results, the EC_50_ values were found to be 0.016 mg/ml and 1.286 mg/ml for WSHA and WSAQ, respectively. The results were compared with the known RT inhibitor AZT (0.49 µM) with more than 90% inhibition of the HIV-1 RT enzyme.

### Molecular docking simulations of phytomolecules against HIV-1 proteins

The phytomolecules from natural sources have significantly impacted human health due to their medicinal potential according to existing literature. Most of these phytomolecules might be good leads as druggable pharmacophores with lesser or even no toxic side effects due to their natural occurrence. During the HIV infection cycle, HIV-1 Integrase, HIV-1 Protease, and HIV-1 Reverse transcriptase proteins are expressed and well-documented in the literature as causative agents of HIV infection. The phytomolecules or the active metabolites, characterized from the root extracts of *Withania somnifera* prepared in hydroalcoholic and aqueous solvent using UHPLC-PDA and mass spectrometry studies, were used for molecular docking simulations [31]. AutoDoc v4.2 was used to understand the inter- as well as intra-molecular interactions between the HIV-1 proteins and the bioactive phytomolecules. The binding energy scores were used to assess the strength of HIV-1 protein-phytomolecule interactions. In docking simulations, all parameters were set to default, however, the protein structures were made rigid and bioactive ligands provided flexibility to interact efficiently. Stable protein–inhibitor complexes were obtained after molecular docking simulations due to molecular interactions between the phytomolecule and residues of the protein. The molecular interactions profile of HIV-1 proteins and the active metabolites are presented in Table 1.

**Table 1:** Molecular interactions profile of HIV-1 proteins (*i.e.*, Integrase, Protease, and Reverse transcriptase) with phytomolecules

Sl. No.	Compound Name	HIV-1 Integrase (PDB: 1QS4)			HIV-1 Protease (PDB: 5KR0)			HIV-1 Reverse transcriptase (PDB: 3QIP)		
		Docking score	Hydrogen bonding interaction	Hydrophobic Interaction	Docking score	Hydrogen bonding interaction	Hydrophobic Interaction	Docking score	Hydrogen bonding interaction	Hydrophobic Interaction
1	12-Deoxywithastramonolide	-7.83	THR66, ASN155	ASP64, HIS67, GLU92, ASP116, GLY118, SER119, ASN120, GLU152, LYS159	-10.72	GLY48, MET46, GLY49, ILE50, ILE54	GLY27, ASP29, ASP30, VAL32, LYS45, GLY51, GLY52, PHE53, PRO81, ILE84	-7.37	GLN161, MET184, VAL381	VAL90, GLN91, GLY93, GLN182, ILE382 and Pi-alkyl (LEU92, ILE94, PRO95, TYR183)
2	27-Hydroxywithanone	-7.75	GLU92, ASN155, LYS159	ASP64, THR66, HIS67, ASP116, GLY118, SER119, ASN120, GLU152, LYS156	-7.97	MET46, GLY48, ILE50, ILE54	ASN25, GLY27, ASP29, ASP30, VAL32, GLY49, GLY52, PHE53, THR80, ILE84	-7.53	VAL90, GLN161, GLN182	TRP88, GLU89, GLN91, GLY93, ILE94, PRO95, THR65, ARG172, MET184
3	Ashwagandhanolide	-5.29	HIS67, GLU92, GLU162, LYS156	ASP64, CYD65, THR66, ASP116, ASN117, GLY118, GLN148, ILE151, ASN155, LYS159	-11.53	LEU24, ASN25, THR26	PRO9, GLY27, ASP29, ASP30, VAL32, GLY48, GLY49, GLY52, PHE53, ILE54, THR80, PRO81, VAL82 and Pi-alkyl (LEU23, ILE47, ILE50)	-11.16	HIS96, ILE94, THR165, ARG172, GLN182	VAL90, GLY93, GLY99, HIS101, GLN161, MET184, VAL381 and Pi-alkyl (PRO95, LEU100, VAL179, THR181, TYR183, ILE382)
4	Withacoagin	-6.49	CYS65, GLN148, ASN155, LYS156	ASP64, THR66, HIS67, ASP116, GLY149, ILE151, GLU152	-10.79	ASP29, GLY48, GLY49, ILE50, ILE54	GLY27, ASP30, VAL32, LYS45, MET46, GLY51, GLY52, PHE53, ILE84	-9.47	VAL90, LEU92, GLY93, GLN161, GLN182	GLN91, ILE94, PRO95, ARG172, ILE180 Pi-alkyl (TRY181, TYR183, MET184)
5	Withaferin A	-7.29	ASN117, LYS156, LYS159	ASP64, CYS65, HIS67, ASP116, GLN148, GLU152, ASN155	-9.65	ASP29, ASP30	ALA28, GLY48, GLY49, ILE50, GLY51, GLY52, PHE53, THR80 PI-ALKYL (VAL32, ILE47, ILE54, LEU76, PRO81, ILE84)	-9.48	LEU92, GLY93, HIS95, GLN161	VAL90, GLN91, ILE94, PRO95, GLY99, TYR183, MET184, VAL381, ILE382 and Pi-alkyl (TYR181)
6	Withanolide A	-6.16	ASP64, HIS67, GLU92, ASN155	CYS65, THR66, ASP116, GLY118, SER119, ASN120, GLN148, GLY149, ILE151, GLU152	-10.75	ASP29, ILE50, GLY48, ILE54	GLY27, ASP30, VAL32, LYS45, MET46, GLY49, GLY52, PHE53, THR80, ILE84, and Pi-alkyl (ALA28, ILE47)	-10.33	VAL90, LEU92, GLY93, GLN161, THR165	GLN91, ILE94, ILE180 and Pi-alkyl (TYR181, TYR183, MET184)
7	Withanolide B	-6.93	CYS65, GLN148	ASP64, THR66, GLU92, ASP116, GLY118, ASN120, GLY149, ILE151, GLU152, ASN155	-10.55	ILE50, ILE54	GLY27, ASP29, ASP30, VAL32, LYS45, MET46, GLY48, GLY49, GLY52, PHE53, THR80, PRO81, ILE84, and Pi-alkyl (ALA28, ILE47)	-11.94	ARG172, ILE180, TYR181	LYS101, TYR188, VQL189, GLY190 and Pi-alkyl (LEU100, VAL106, VAL179)
8.	Withanone	-6.77	ASP64, THR66, LYS159	CYS65, HIS67, ASP116, GLN148, GLY149, GLU152, ASN155, LYS156	-10.02	GLY48, ILE50	GLY27, ASP29, ASP30, VAL32, LYS45, GLY52, PHE53, THR80, PRO81 PI-ALKYL (ALA48, ILE47)	-7.8	GLN182	LEU92, GLY93, ILE94, PRO95, GLN161, THR165, ARG172, ILE180, MET184 and Pi-alkyl (VAL90, TYR183)
9.	Withanoside IV	-2.01	ASP64, HIS67, GLU92, ASN117	CYS65, ASP116, GLY118, PHE121, GLN148, GLU152, ASN155	-5.75	ASP30	IEU23, ASN25, ALA28, ASP29, GLY27, GLY48, GLY49, GLY52, ILE54, LEU76, PRO81 PI-ALKYL (VAL32, ILE47, ILE50, ILE84)	-6.14	VAL90, LEU92, GLY93, LY99, LYS101, VAL381, GLY384	GLN91, ILE94, LEU100, GLN161, GLN182, MET184, ILE300, ILE382, TRP383 and Pi-alkyl (PRO95, TYR183)
10.	Withanoside V	-3.15	ASP64, THR66, ASP116, GLN148, LYS159	CYS65, HIS67, ILE151, GLU152, ASN155	-7.66	ASP29, ASP30, GLY48, LYS45	ALA28, ASP30, VAL32, LYS47, GLY48, GLY49, ILE50, THR80, PRO81, ILE84	-10.13	VAL90, LEU92, GLY93, GLN161	ILE94, PRO95, LEU100, LYS103, VAL106, TYR188, ILE180, TYR181, VAL381

### Molecular interaction between HIV-1 Integrase and 12-Deoxywithastramonolide & 27-Hydroxywithanone

**Fig. 6:**
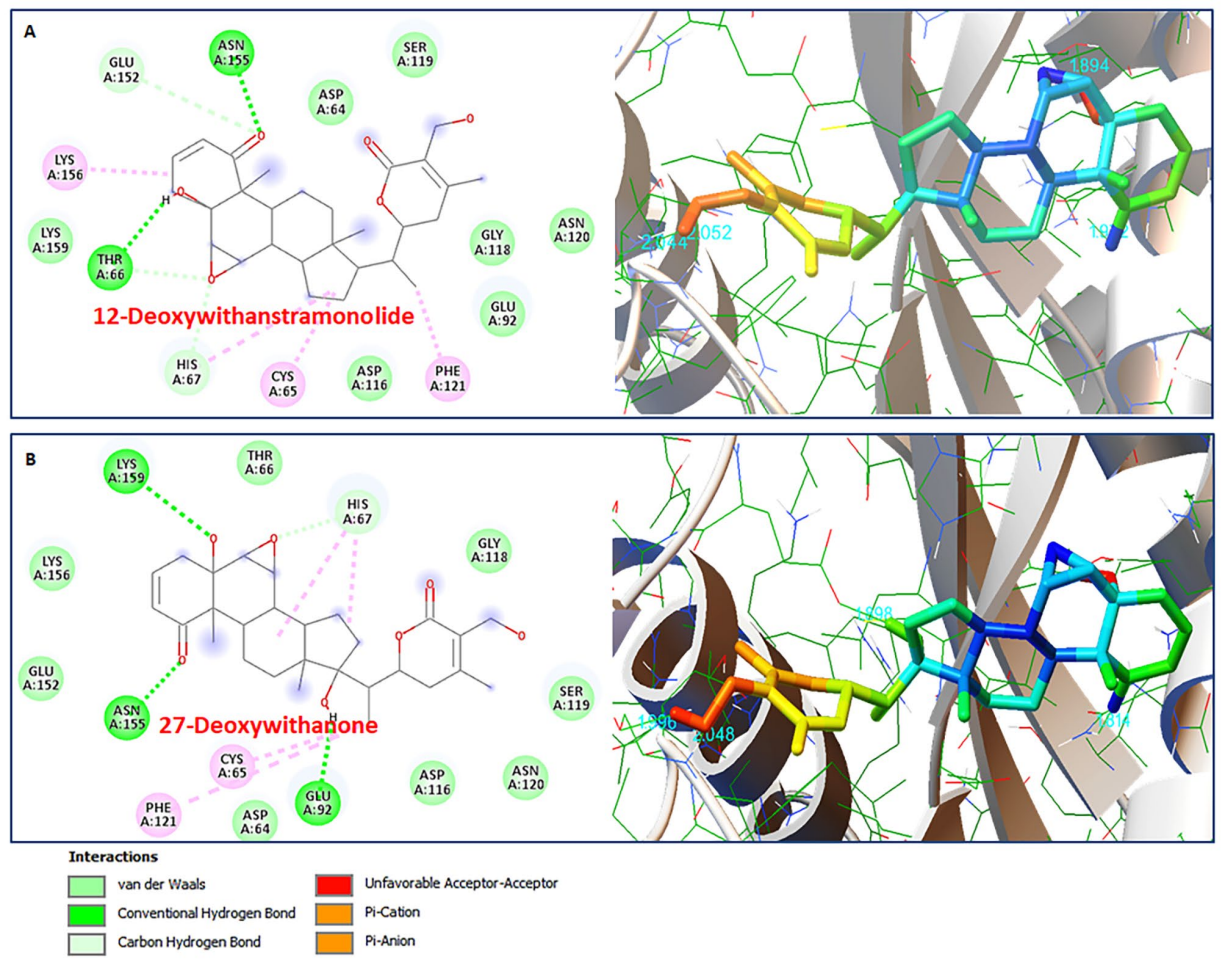
Molecular interactions between HIV-1 Integrase and phytomolecules of *Withania somnifera*. 2D interaction plot (left panel) and 3D map (right panel) of molecular docking simulaiton after (**A**) 12-Deoxywithastramonolide (CID:44576309) and (**B**) 27-Hydroxywithanone (CID:21574483) as residue contacts against HIV-1 Integrase (PDB:1QS4) activity

HIV-1 Integrase is one of the essential enzymes involved in the retrovirus’ replication cycle and it stimulates the integration of viral DNA that has undergone Reverse transcription into chromosomal DNA. Such integration of HIV-1 DNA ensures the stability of the viral genome, hence, the virus continues to exist in the host [37]. In the present study, the molecular docking analysis of HIV-1 Integrase (PDB: 1QS4) with 12-Deoxywithastramonolide and 27-Hydroxywithanone revealed low binding energies of -7.83 Kcal/mol and -7.75 Kcal/mol, respectively, for molecular conformations induced by the phytomolecules (Table 1). 12-Deoxywithastramonolide established two hydrogen bonds with THR66, and ASN155 within 4Å cut-off and nine hydrophobic interactions with ASP64, HIS67, GLU92, ASP116, GLY118, SER119, ASN120, GLU152, and LYS159 (Fig. 6A), whereas, 27-Hydroxywithanone established three hydrogen bonds with GLU92, ASN155, and LYS159 residues within 4Å cut-off and nine hydrophobic interactions with ASP64, THR66, HIS67, ASP116, GLY118, SER119, ASN120, GLU152, and LYS156 residues during molecular docking simulations in the vicinity of the active site binding pocket responsible for the catalytic activity of HIV-1 Integrase (Fig. 6B).

**Fig. 7:**
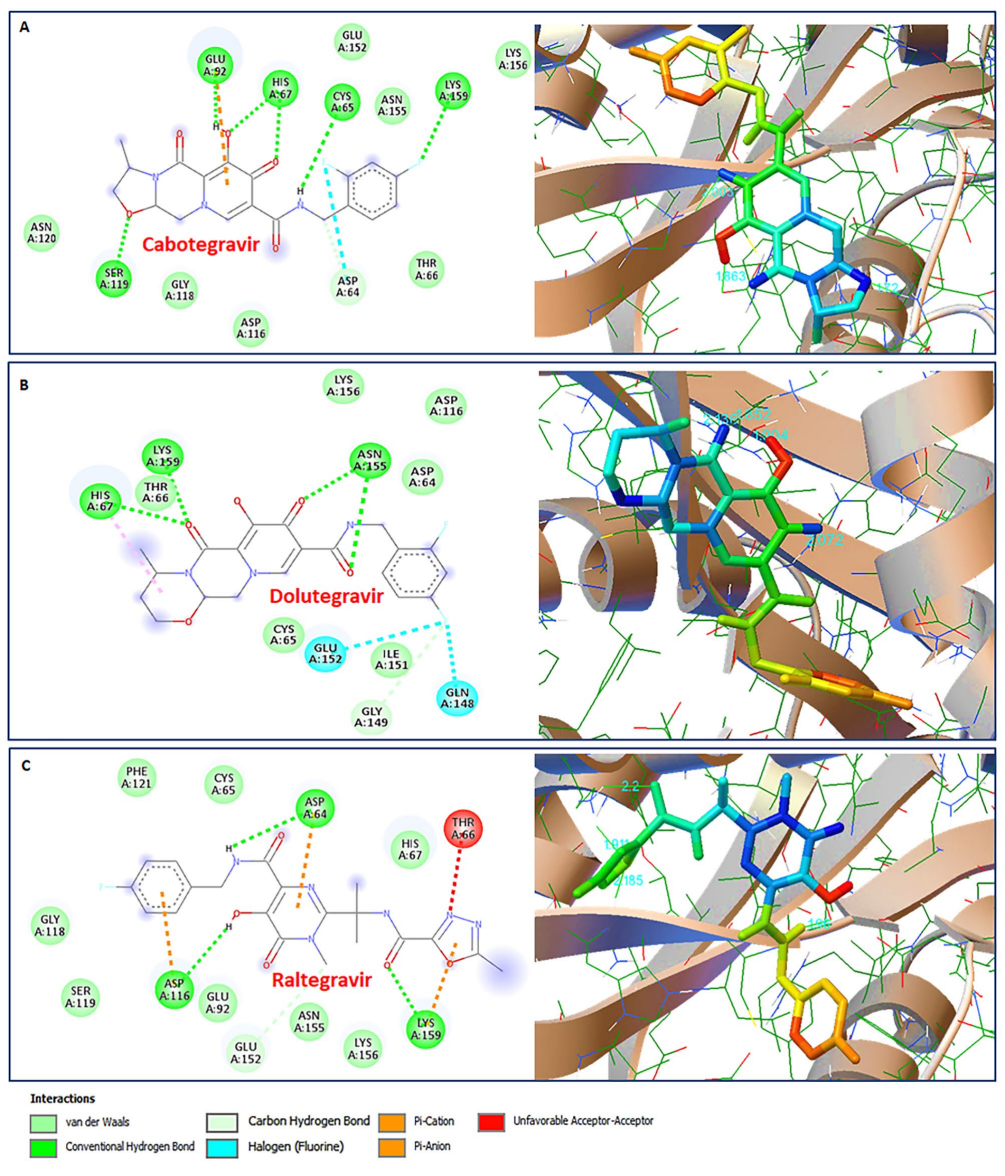
Molecular docking simulation analysis of HIV Integrase (PDB:1QS4) with FDA approved drugs in the active binding site. 2D interaction plot (left panel) and 3D map (right panel) of (**A**) Cabotegravir (CID:54713659), (**B**) Dolutegravir (CID:46216142) and (**C**) Raltegravir (CID:23668479).

The molecular docking simulations interaction analyses for the known FDA approved HIV-1 Integrase inhibitors, *viz.* Cabotegravir, Dolutegravir and Raltegravir were presented in Table 2. Cabotegravir was able to establish only five hydrogen bond with CYS65, HIS67, GLU92, SER119, and LYS159 within 4Å cut-off and eight hydrophobic interactions with ASP64, THR66, ASP116, GLY118, ASN120, GLU152, ASN155, and LYS156 in the active binding pocket residues of HIV-1 Integrase (Fig. 7A), however, Dolutegravir established three hydrogen bonds with HIS67, LYS159, and ASN155 within 4Å cut-off and six hydrophobic interactions with ASP64, CYS65, THR66, ASP116, ILE151, and LYS156 (Fig. 7B). Furthermore, Raltegravir established three hydrogen bonds with ASP64, ASP116, and LYS159 within 4Å cut-off and ten hydrophobic interactions with CYS65, HIS67, GLU92, GLY118, SER119, PHE121, GLU152, ASN155, and LYS156 (Fig. 7C). Interestingly, the lower binding energy of -7.83 Kcal/mol for 12-Deoxywithastramonolide and  -7.75 Kcal/mol for 27-Hydroxywithanone emphasizes strong binding capabilities to inhibit HIV-1 Integrase activity and higher than the FDA approved drugs (Table 2).

**Table 2:** Molecular interactions profile of HIV-1 Integrase (PDB:1QS4), Protease (PDB:5KR0), and Reverse transcriptase (PDB:3QIP) with FDA approved drugs after docking

Sl. No.	Drug Molecule	Binding Energy (Kcal/Mol)	Inhibition constant (µM)	Hydrogen Bond interaction	Hydrophobic bond interaction
1	Cabotegravir	-5.21	150.46	CYS65, HIS67, GLU92, SER119, LYS159	ASP64, THR66, ASP116, GLY118, ASN120, GLU152, ASN155, LYS156
2	Dolutegravir	-5.82	54.19	HIS67, LYS159, ASN155	ASP64, CYS65, THR66, ASP116, ILE151, LYS156
3	Raltegravir	-5.18	160.43	ASP64, ASP116, LYS159	CYS65, HIS67, GLU92, GLY118, SER119, PHE121, GLU152, ASN155, LYS156
4	Ritonavir	-4.35	645.16	ASN25, ILE50	LEU24, THR26, GLY27, VAL32, GLY48, PHE53, PRO79, THR80, VAL82, ILE84
5	Zidouvdine	-7.23	5.0	LYS101, HIS235	LYS102, VAL179, GLY190, PHE227, PRO236, ASP237, LYS238

*In silico* analysis also elucidated that phytomolecules from *Withania somnifera*, such as Withanolide B (-6.93Kcal/mol), Withanone (-6.77 Kcal/mol), Withacoagin (-6.49 Kcal/mol), Withaferin A (-7.29 Kcal/mol), and Withanolide A (-6.16 Kcal/mol) could be responsible for inhibition of HIV-1 Integrase precisely, as evidenced from the binding energy analysis, hydrogen bond, and hydrophobic bond interactions (Table 1 and Figure S1).

### Molecular interaction between HIV-1 Protease and Ashwagandhanolide & Withacoagin

**Fig. 8:**
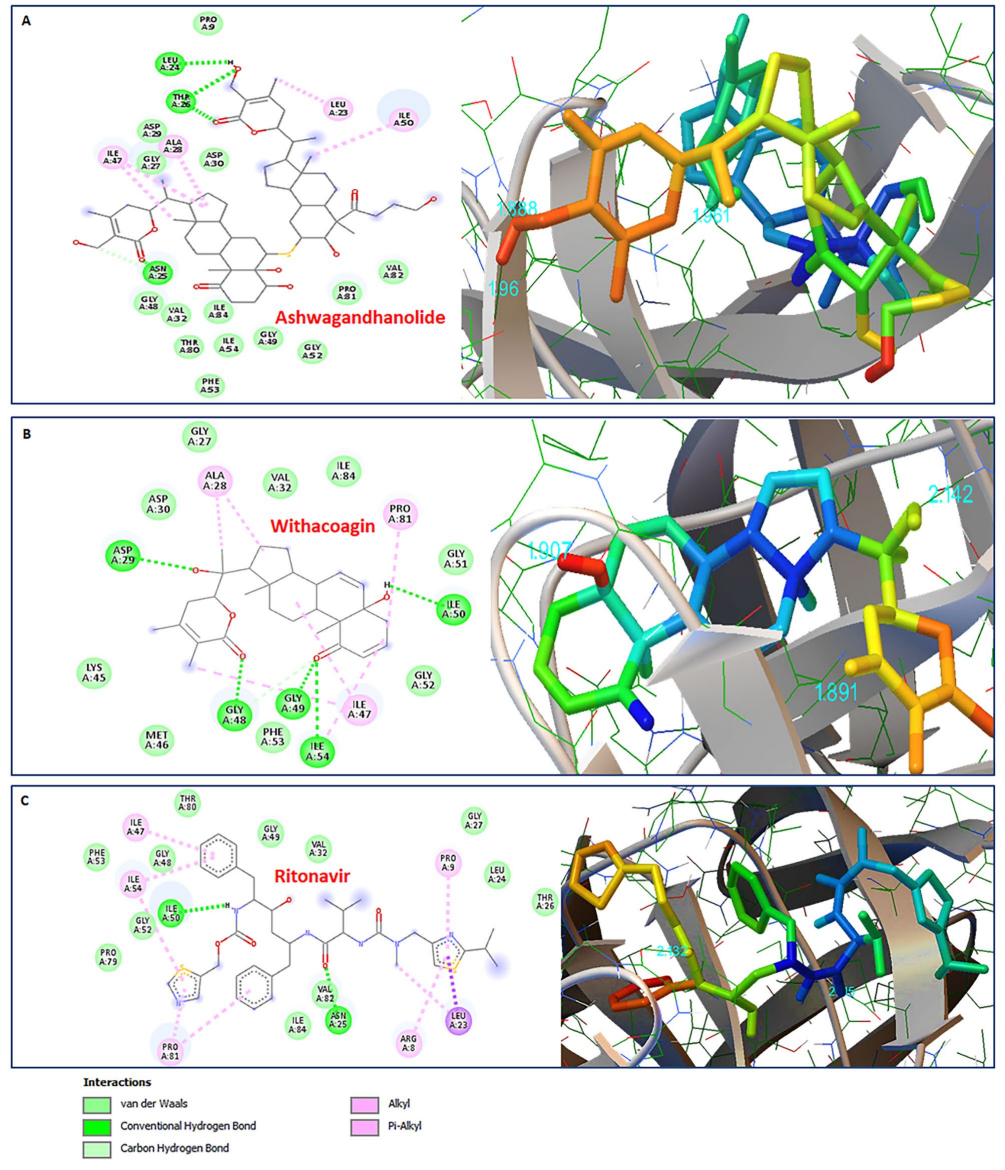
Molecular interactions between HIV-1 Protease and phytomolecules of *Withania somnifera*. 2D interaction plot (left panel) and 3D map (right panel) of molecular docking simulaiton after (**A**) Ashwagandhanolide (CID:16099532) and (**B**) Withacoagin (CID:14236709) as residue contacts against HIV-1 Protease (PDB:5KR0) activity. (**C**) Molecular docking simulation analysis of FDA approved known protease inhibitor Ritonavir (CID:392622) with HIV-1 Protease (PDB:5KR0) in the active binding site depicted in 2D interaction plot (left) panel and 3D map (right panel)

HIV-1 Protease is crucial for HIV replication and maturation into an infectious form. Therefore, one of the main focus areas for HIV-1 drug development efforts is primarily the inhibition of this enzyme [38]. The HIV-1 Protease (PDB:5KR0) activity may also be inhibited by the phytomolecules of *Withania somnifera* as identified through the *in silico* molecular interactions in the present docking simulations study. Out of the ten such phytomolecules, Ashwagandhanolide (-11.53 Kcal/mol) and Withacoagin (-10.79 Kcal/mol) were identified as the lead molecules, however, others were also found effective by binding in the active binding pocket of HIV-1 Protease. Ashwagandhanolide established three hydrogen bonds with LEU24, ASN25, and THR26 within 4Å cut-off and thirteen hydrophobic bond interactions with PRO9, GLY27, ASP29, ASP30, VAL32, GLY48, GLY49, GLY52, PHE53, ILE54, THR80, PRO81, VAL82 and three Pi-alkyl bonds with LEU23, ILE47, and ILE50 residues present in the active binding pocket (Table 1; Fig. 8A). Additionally, Withacoagin established molecular interactions with HIV-1 Protease residues *via* five hydrogen bonds with ASP29, GLY48, GLY49, ILE50, and ILE54 within 4Å cut-off and nine hydrophobic interactions with GLY27, ASP30, VAL32, LYS45, MET46, GLY51, GLY52, PHE53, ILE84 residues available in binding pocket (Table 1; Fig. 8B). The *in silico* analysis was also performed on FDA approved HIV-1 Protease inhibitor Ritonavir. Interestingly, Ritonavir was able to establish only two hydrogen bonds with ASN25 and ILE50 within 4Å cut-off and ten hydrophobic interactions with LEU24, THR26, GLY27, VAL32, GLY48, PHE53, PRO79, THR80, VAL82, and ILE84 residues of HIV-1 Protease active binding pocket (Table 2; Fig. 8C). The molecular docking simulations were further extended to evaluate other phytomolecules of *Withania somnifera* against HIV-1 Protease. The phytomolecules *viz.*, 27-Hydroxywithanone (-7.97 Kcal/mol), Withanolide A (-10.75 Kcal/mol), 12-Hydroxywithastramonolide (-10.72), Withanolide B (-10.55 Kcal/mol), and Withaferin A (-9.65 Kcal/mol) interacted with HIV-1 Protease showing promising binding energies, forming many hydrogen bonds within 4Å cut-off and hydrophobic bond interactions with active binding site pocket residues (Table 1 and Figure S2). All the above-mentioned residues occupied by these phytomolecules are responsible for the activation of HIV-1 Protease, however, this occupancy renders the inhibitory effect on HIV replication.

### Molecular interaction between HIV-1 Reverse transcriptase and Ashwagandhanolide & Withanolide B

**Fig. 9:**
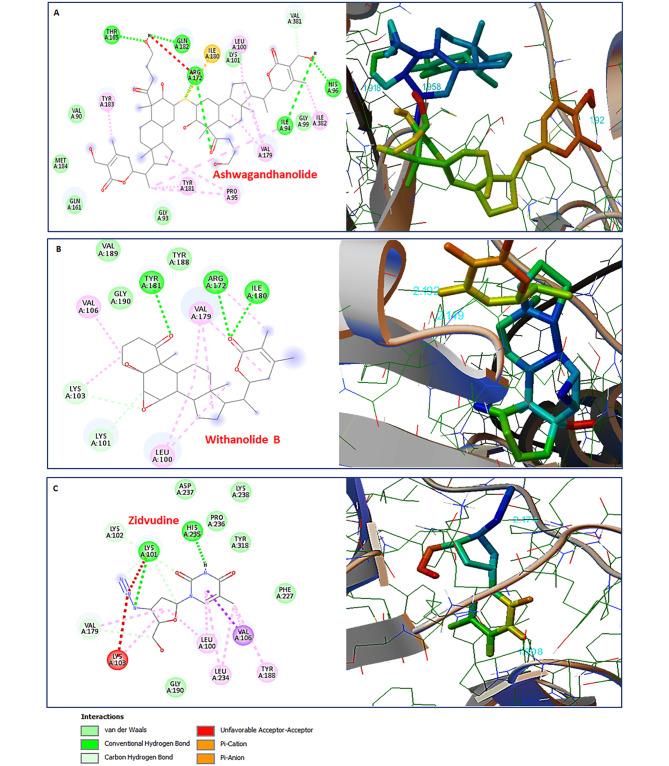
Molecular interactions between HIV-1 Reverse transcriptase and phytomolecules of *Withania somnifera*. 2D interaction plot (left panel) and 3D map (right panel) of molecular docking simulaiton after (**A**) Ashwagandhanolide (CID:16099532) and (**B**) Withnoalide B (CID:14236711) as residue contacts against HIV-1 RT (PDB:3QIP) activity. (**C**) Molecular docking simulation analysis of FDA approved known HIV-1 RT inhibitor Zidovudine (CID:35370) with HIV-1 RT (PDB:3QIP) in the active binding site depicted in 2D interaction plot (left) panel and 3D map (right panel)

The HIV-1 Reverse transcriptase or RT is a unique and essential enzyme that cooperates in the reverse transcription step of HIV-1 replication. The HIV-1 RT (PDB:3QIP) activity may also be inhibited by the phytomolecules Ashwagandhanolide and Withanolide B, as they were ranked the best among all the tested phytomolecules of *Withania somnifera*. Ashwagandhanolide exhibited  -11.16 Kcal/mol binding energies interacting with HIV-1 Reverse transcriptase *via* forming five hydrogen bonds with HIS96, ILE94, THR165, ARG172, and GLN182 residues within a 4 Å cut-off and seven hydrophobic bond interactions with VAL90, GLY93, GLY99, HIS101, GLN161, MET184, and VAL381 residues and six Pi-alkyl bonds with PRO95, LEU100, VAL179, THR181, TYR183, and ILE382 residues found in the active site (Table 1; Fig. 9A). Whereas, Withanolide B found molecular interactions with HIV-1 RT *via* forming three hydrogen bonds with ARG172, ILE180, and TYR181 within a 4 Å cut-off along with four hydrophobic interactions with LYS101, TYR188, VQL189, and GLY190 residues. Moreover, three Pi-alkyl interactions with LEU100, VAL106, and VAL179 were also established with the binding pocket residues of HIV-1 RT by Withanolide B (Table 1; Fig. 9B). Withanolide B was found to be the top ranking phytomolecule that exhibited the least binding energy of -11.94 Kcal/mol in comparison to any other phytomolecules found during this study (Table 1).

These residues are part of the active binding pocket of HIV-1 RT and are responsible for the catalytic mechanism. The occupancy of these important residues by Ashwagandhanolide and Withanolide B might hinder the opportunity for HIV-1 viral protein to replicate. The HIV-1 RT activity governed by these important residues might also be impeded as they were occupied by many phytomolecules, for example Withanolide A (-10.33 Kcal/mol), Withanoside V (-10.13 Kcal/mol), Withaferin A (-9.48 Kcal/mol), Withacoagin (-9.47 Kcal/mol), Withanone (-7.8 Kcal/mol), 27-Hydroxywithanone (-7.53 Kcal/mol), and 12-Deoxywithastramonolide (-7.37 Kcal/mol) (Table 1 and Figure S3). These phytomolecules successfully established hydrogen and hydrophobic bond interactions with many important residues found in the active binding pocket of HIV-1 RT and might be responsible for the inhibition of HIV-1 replication, therefore restricting the viral infection.

A control *in silico* experiment with FDA approved HIV-1 Reverse transcriptase inhibitor, Zidouvdine, was performed for molecular interaction comparison. It is quite interesting to note that Zidouvdine bound with HIV-1 RT with a binding energy of -7.23 Kcal/mol which was still far less than most of the phytomolecules of *Withania somnifera* (Table 2; Fig. 9C). Molecular interaction analysis also revealed that Zidouvdine established only two hydrogen bonds with LYS101 and HIS235 within a 4Å cut-off and only seven hydrophobic interactions with LYS102, VAL179, GLY190, PHE227, PRO236, ASP237, and LYS238 residues (Fig. 9C).

### *In silico* prediction of ADMET properties of phytomolecules

The pharmacokinetics and drug-likeliness potential of the ten phytomolecules were predicted using the Swiss-ADME, cheminformatics platform. Bioavailability RADAR analysis showed that Withacoagin, Withaferin A, Withanone, Withanolide A, and Withanolide B are orally bioavailable except Ashwagandhanolide, Withanoside IV, and Withanoside V (Table 3). The ADMET properties also reveal that the phytomolecules selected in this study will be the substrate of the P-glycoprotein transporter (Table 3). P-glycoprotein is a part of the ATP-binding cassette (ABC) transporter, therefore, the answer “yes” for the P-glycoprotein substrate denotes that all phytomolecules are predicted to be transported across the cell membrane by the ABC transporter. However, none of the molecules has shown an ability to penetrate the blood-brain barrier (BBB). The measured skin permeation coefficient (Log Kp cm/s) for all phytomolecules with a value of ≤ -2.5 suggests poor skin permeability due to high molecular size and lipophilicity. However, all the phytomolecules can be well absorbed into the body of the patients.

**Table 3:** *In silico* ADMET properties of phytomolecules

Sl. no.	Phytomolecule	Druggability (Lipinski’s rule of five)	Pharmacokinetics properties				
			GI Absorption	BBB Permeate	P-gp Substrate	CYP	Log Kp (Skin permeation)
1	12-Deoxywithastramonolide	Yes	High	No	Yes	CYP2C9	-6.35 cm/s
2	27-Hydroxywithanone	Yes	High	No	Yes	No	-7.60 cm/s
3	Ashwagandhanolide	No	Low	No	Yes	CYP3A4	-6.95 cm/s
4	Withacoagin	Yes	High	No	Yes	CYP2C9	-6.29 cm/s
5	Withaferin A	Yes	High	No	Yes	No	-6.45 cm/s
6	Withanolide A	Yes	High	No	Yes	No	-6.86 cm/s
7	Withanolide B	Yes	High	No	Yes	CYP2C9	-5.76 cm/s
8	Withanone	Yes	High	No	Yes	No	-7.01 cm/s
9	Withanoside IV	No	Low	No	Yes	No	-10.37 cm/s
10	Withanoside V	No	Low	No	Yes	No	-9.79 cm/s

The results of the docking interaction of phytomolecules with the HIV-1 proteins (*i.e.*, Integrase, Protease and Reverse transcriptase) revealed low binding energies, which means the selected phytoconstituents might establish good atomic interactions with the active binding pocket residues. Furthermore, the structural properties of these selected phytomolecules of *Withania Somnifera* are presented in Table 4.

**Table 4:** Structural properties of phytomolecules

Sl. no.	Phytomolecule	Descriptors					
		Surface Area (Å2 )	Molecular Weight (g/mol)	LogP	Rotatable Bonds	Acceptors	Donors
1	12-Deoxywithastramonolide	96.36	470	3.14	3	6	2
2	27-Hydroxywithanone	116.59	486.60	3.20	3	7	3
3	Ashwagandhanolide	411.756	975.295	6.3768	8	13	6
4	Withacoagin	83.83	454.60	3.88	2	5	2
5	Withaferin A	96.36	470.60	3.24	3	6	2
6	Withanolide A	96.36	470.60	3.68	2	6	2
7	Withanolide B	76.13	454.60	3.54	2	5	1
8	Withanone	96.36	470.60	3.46	2	6	2
9	Withanoside IV	245.29	782.91	0.68	9	15	9
10	Withanoside V	225.06	766.91	1.36	8	14	8

## Discussion

**Fig. 10:**
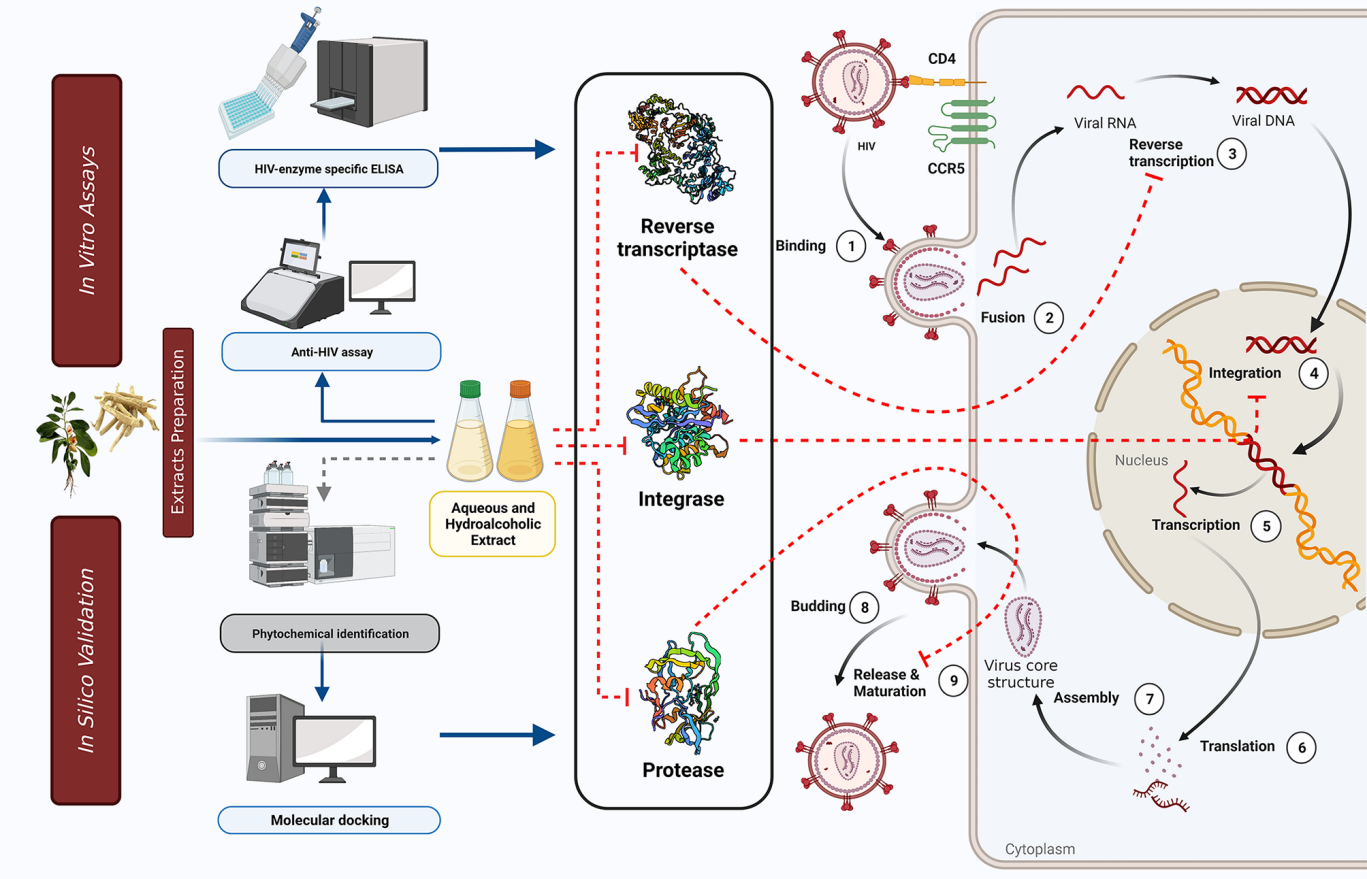
Simplified schematic illustration based on our in vitro and *in silico* findings depicting the possible sites of intervention of the HIV-1 replication cycle. The WSHA and WSAQ extracts and their phytomolecules were effective as inhibitors of/against the viral replication cycle enzymes *viz.* Integrase, Reverse transcriptase, and Protease. The red dotted line represents the inhibitory effect of extracts on HIV-1 life cycle, blue solid line depicts the schematic work flow of the study, and grey dotted arrow indicating data from our previous study [31]

Even though *Withania somnifera* or Ashwagandha is most frequently used in Indian Ayurvedic medicine, its bioactive components’ potential and mechanistic effects on HIV-1 infection have never been explored [39]. Hence, in this study, we focused on the anti-HIV-1 activity, mode of action, and the role of phytomolecules present in *Withania somnifera* extracts. We tried to unveil the anti-HIV-1 potential of hydroalcoholic and aqueous extraction of *Withania somnifera* through cell-based assays, mechanistic validations by the enzymatic analyses, and finally through the *in silico* interaction studies by molecular docking and simulation. In brief, our experimental data indicate (1) the safety profile and anti-HIV-1 potential of *Withania somnifera* hydroalcoholic and aqueous extracts, (2) the mode of action of extracts through multi-target activities, might be acted on HIV-1 Integrase, Protease, and Reverse Transcriptase inhibition, which is inferred through the time of addition cell-based and enzymatic assays, and (3) at least ten key phytomolecules of *Withania somnifera* are identified as potential anti-HIV-1 agents. All-in-all, the data suggest that the WSHA and WSAQ extracts and their phytomolecules are well capable of inhibiting HIV-1 replication (Fig. 10).

The clinical safety profile of WSAQ has already been investigated earlier. All of the healthy volunteers, included in the previous study tolerated a dose of 625 mg twice a day without experiencing any toxic effects [40]. Through an in vivo study, WSHA extract was reported with the sign of no toxicity as well [41]. Likewise, our results also showed that the extracts of WSHA and WSAQ are safe up to ≤ 0.252 mg/ml and ≤ 1.987 mg/ml in both TZM-bl cell lines and human PBMCs (Fig. 1).

Several in vitro studies and literary reports suggest that crude extracts of *Withania somnifera* and its constituents are enriched with phytochemicals and known to possess anti-viral activity including SARS-CoV-2, Herpes Simplex Virus-1, Chikungunya virus, H1N1 influenza virus infections [31, 42–49]. In support of the previous findings, our results also indicate that the *Withania somnifera* extracts have anti-retroviral potential against X4 and R5 clades of HIV-1 (Figs. 2 and 3). Further, we also carried out the time-of-addition (TOA) experiment to determine its mechanism of action (Fig. 4). This TOA determines the in vitro stability of a compound without losing its antiviral properties. When compared to a reference drug, an antiviral compound’s relative location in the time scale can be used to determine the target. Once an unknown drug’s profile resembles that of an existing anti-HIV drug, the unknown drug is likely targeting through the same mechanism, or at the very least one that is active at the same time [34]. A previous study demonstrated the mechanism of action in the HIV-1 replication cycle comparable to a known inhibitor [50]. Similarly, the in vitro findings that WSAQ exhibited an HIV-1 Protease inhibition pattern similar to RTV and WSHA showed an inhibition pattern similar to the known Integrase inhibitor RAL, respectively, indicating the mode of action of these extracts (Fig. 4). The advantage of this method is that it establishes the potential target of an inhibitor’s interaction and serves as the starting point for additional research with just one experiment; however, the limitation of this assay is that the results from compounds with limited selectivity are difficult to interpret because they depend on the fold of concentrations. This can be explained by both the extent of cellular uptake of the drug(s) at different concentrations and their differential potency against multiple processes [34]. Conversely, enzymatic assays are more accurate for determining the activity of each inhibitor or extract against HIV-1; hence, we confirmed our results using the enzymatic assays.

In continuation of efforts in search for new antivirals against HIV-1, we performed *in silico* interaction studies of phytoconstituents as ligands against HIV-1 targets. Our previous study has identified and characterized the phytoconstituents of the *Withania somnifera* and reported its anti-COVID-19 potential [31]. HIV-1 Integrase, Protease, and Reverse transcriptase are the three vital proteins that power the molecular motor to propel viral replication. *In silico* analysis elucidates that phytomolecules from *Withania somnifera*, such as Ashwagandhanolide, Withacoagin, Withaferin A, Withanolide A, Withanolide B, Withanone, and Withanone, which exhibited the lower binding energy scores, hence, could be the potent inhibitors of HIV-1 Integrase (Table 1). The sum of interactions between the pair of ligand and receptor pharmacophores determines the receptor-ligand binding energy. When a drug molecule binds to a specific target, the released binding energy lowers the complex’s overall stability. Hence, the lesser energy released after the binding of a ligand to its receptor protein, the greater will be the propensity of that ligand-protein association. Interestingly, the lowest binding energy of -7.83 Kcal/mol for 12-Deoxywithastramonolide and  -7.75 Kcal/mol for 27-Hydroxywithanone emphasizes strong binding capabilities to inhibit HIV-1 Integrase activity and even higher than the FDA approved drugs (Figs. 6 and 7). It is important to note that, out of almost 40 HAART (Highly active antiretroviral therapy) drugs approved by the US Food and Drug Administration (FDA), only three HIV-1 Integrase targets, *viz.* Cabotegravir, Dolutegravir, and Raltegravir, are available (https://hivinfo.nih.gov/understanding-hiv/fact-sheets/fda-approved-hiv-medicines). 12-Deoxywithastramonolide and 27-Hydroxywithanone established two and three hydrogen bonds, respectively, in the vicinity of the active binding pocket of HIV-1 Integrase (Fig. 6; Table 1), however, Cabotegravir established five hydrogen bonds and Dolutegravir or Raltegravir succeeded to establish three hydrogen bonds, as identified by the molecular docking simulation analyses (Fig. 7; Table 2). Furthermore, these results were also supported by the in vitro enzymatic assays of WSHA and WSAQ extracts, which exhibited more than 85% inhibition in both extracts (Fig. 5A and B). This is the first report of HIV-1 Integrase inhibition by the root extracts of *Witahnia Somnifera*. In concurrence with our findings, the effects of natural compounds like *Punica granutum* extracts have shown anti-HIV-1 Integrase properties previously [51]. Thus, screening of novel anti-HIV-1 Integrase inhibitor drug candidates with improved potency, pharmacokinetic profiles and minimal side effects is the need of the hour.

Similarly, Ashwagandhanolide (-11.53 Kcal/mol) and Withacoagin (-10.79 Kcal/mol) were identified as the lead molecules, however, others were found effective by interacting with the active binding pocket of HIV-1 Protease. Based on the binding energy values, these two phytomolecules could bind to the active residues of HIV-1 Protease stronger than the other ligands, however, the rest of the phytomolecules were also found effective by establishing bond in the active binding pocket of HIV-1 Protease (Fig. 8, Figure S2 and Table 1). The in vitro results revealed more than 50% inhibition of HIV-1 Protease activity even at the lower dosages of half maximal effective concentrations (EC_50_) of WSHA and WSAQ extracts (Fig. 5C and D). Our results are consistent with the previous findings where the extracts and phytoconstituents of a plant *Alpinia galanga* of Zingiberaceae displayed the inhibitory activity on HIV-1 Protease [52].

*In silico* results revealed that phytomolecules of *Withania Somnifera* showed strong binding affinity to the HIV-1 RT active sites in comparison to the Integrase or Protease. Ashwagandhanolide exhibited  -11.16 Kcal/mol binding energies interacting with HIV-1 Reverse transcriptase *via* forming five hydrogen bonds, whereas, Withanolide B exhibited  -11.94 Kcal/mol binding energies and found molecular interactions with HIV-1 RT via forming three hydrogen bonds (Fig. 9; Table 1). However, the in vitro activities of the extracts exhibited moderate inhibition against HIV-1 RT by 76.82% and 58.53% even at the highest sub-cytotoxic concentrations of WSHA and WSAQ, respectively (Fig. 5E and F). The potential of the Ayurvedic plant, *Hemidesmus indicus*, was reported earlier as a valuable multi-target active drug source by virtue of numerous active metabolites [53]. Numerous studies showed that Withanolide (steroidal lactones), the main phytochemical of *Withania Somnifera*, exhibited multimodal synergistic effects, and played a central role against several disease conditions [6, 54, 55]. Similarly, the findings from this study also indicated 12-Deoxywithastramonolide, 27-Hydroxywithanone, Ashwagandhanolide, Withacoagin, and Withanolide B as the key components of *Withania Somnifera* that might have multi-target activity in the extenuation of HIV-1 infection.

However, further explorative research is required through in vitro and in vivo assessment of each phytomolecules, individually, as well as in combination to scrutinize the therapeutic levels against HIV-1 infection. It’s plausible that additional cell-free anti-viral mechanisms could also be involved in the inhibition of HIV-1 replication and are also required to be investigated in our future ventures.

## Conclusions

In conclusion, this study reports the significant anti-HIV-1 activity of hydroalcoholic and aqueous extracts of *Withania somnifera*. Furthermore, using in vitro mechanistic analyses and *in silico* molecular interaction studies involving the key viral proteins, we were able to identify the suppression of the three crucial HIV-1 enzymes, Integrase, Protease, and Reverse transcriptase. Our findings also revealed 12-Deoxywithastramonolide, 27-Hydroxywithanone, Ashwagandhanolide, Withacoagin, and Withanolide B as the key elements of *Withania somnifera* with the highest binding affinities against the HIV-1 proteins, denoting the possible mechanism of action in limiting HIV-1 infection. Overall, these results indicate the significant role of *Withania somnifera* extracts as a potential source of multi-target active drugs against HIV-1 infection. To prevent the use of several medications with various activities and multiple side effects, this study might lead to a special interest in the key elements that can be used in the development of derivatives with multifaceted functions to control HIV-1 infection.

### Electronic supplementary material

Below is the link to the electronic supplementary material.

Supplementary Material 1

Supplementary Material 2

**Figure S1**. Molecular docking simulation analysis of HIV Integrase (PDB: 1QS4), with natural phytomolecules in *Withania somnifera*, in the active binding site. 2D interaction plot (left panel) and 3D map (right panel) of (A) Withanolide B (CID: 14236711), (B) Withanone (CID: 21679027), (C) Withacoagin (CID: 14236709), (D) Withaferin A (CID: 265237), and (E) Withanolide A (CID: 11294368). **Figure S2**. Molecular docking simulation analysis of HIV Protease (PDB: 5KR0), with natural phytomolecules in *Withania somnifera*, in the active binding site. Left panel (2D interaction plot) and right panel (3D map) (A) 27-Hydroxywithanone (CID: 21574483), (B) Withanolide A (CID: 11294368), (C) 12-Deoxywithastramonolide (CID: 44576309), (D) Withanolide B (CID: 14236711), (E) Withanone (CID: 21679027), and (F) Withaferin A (CID: 265237). **Figure S3**. Molecular docking simulation analysis of HIV-1 Reverse Transcriptase (PDB: 3QIP) with natural phytomolecules from *Withania somnifera* and FDA approved drug Zidovudine in the active binding site. Left panel (2D interaction plot) and right panel (3D map) (A) Withanolide A (CID: 11294368), (B) Withanoside V (CID: 10700345), (C) Withaferin A (CID: 265237), (D) Withacoagin (CID: 14236709), (E) Withanone (CID: 21679027), (F) 27-Hydroxywithanone (CID: 21574483), and (G) 12-Deoxywithastramonolide (CID: 44576309).

## Data Availability

All data generated or analysed during this study are included in this published article [and its supplementary information files].
