# Supplementary material for: Withania somnifera extracts induced attenuation of HIV-1: a mechanistic approach to restrict viral infection
Source: Virol J. 2023 Aug 3;20:173. doi: 10.1186/s12985-023-02130-y (PMC10401819; doi:10.1186/s12985-023-02130-y)
Supplement: Supplementary file 3 — Figure S1. Molecular docking simulation analysis of HIV Integrase (PDB: 1QS4), with natural phytomolecules in Withania somnifera, in the active binding site. 2D interaction plot (left panel) and 3D map (right panel) of (A) Withanolide B (CID: 14236711), (B) Withanone (CID: 21679027), (C) Withacoagin (CID: 14236709), (D) Withaferin A (CID: 265237), and (E) Withanolide A (CID: 11294368). Figure S2. Molecular docking simulation analysis of HIV Protease (PDB: 5KR0), with natural phytomolecules in Withania somnifera, in the active binding site. Left panel (2D interaction plot) and right panel (3D map) (A) 27-Hydroxywithanone (CID: 21574483), (B) Withanolide A (CID: 11294368), (C) 12-Deoxywithastramonolide (CID: 44576309), (D) Withanolide B (CID: 14236711), (E) Withanone (CID: 21679027), and (F) Withaferin A (CID: 265237). Figure S3. Molecular docking simulation analysis of HIV-1 Reverse Transcriptase (PDB: 3QIP) with natural phytomolecules from Withania somnifera and FDA approved drug Zidovudine in the active binding site. Left panel (2D interaction plot) and right panel (3D map) (A) Withanolide A (CID: 11294368), (B) Withanoside V (CID: 10700345), (C) Withaferin A (CID: 265237), (D) Withacoagin (CID: 14236709), (E) Withanone (CID: 21679027), (F) 27-Hydroxywithanone (CID: 21574483), and (G) 12-Deoxywithastramonolide (CID: 44576309). [file 12985_2023_2130_MOESM3_ESM.pdf]

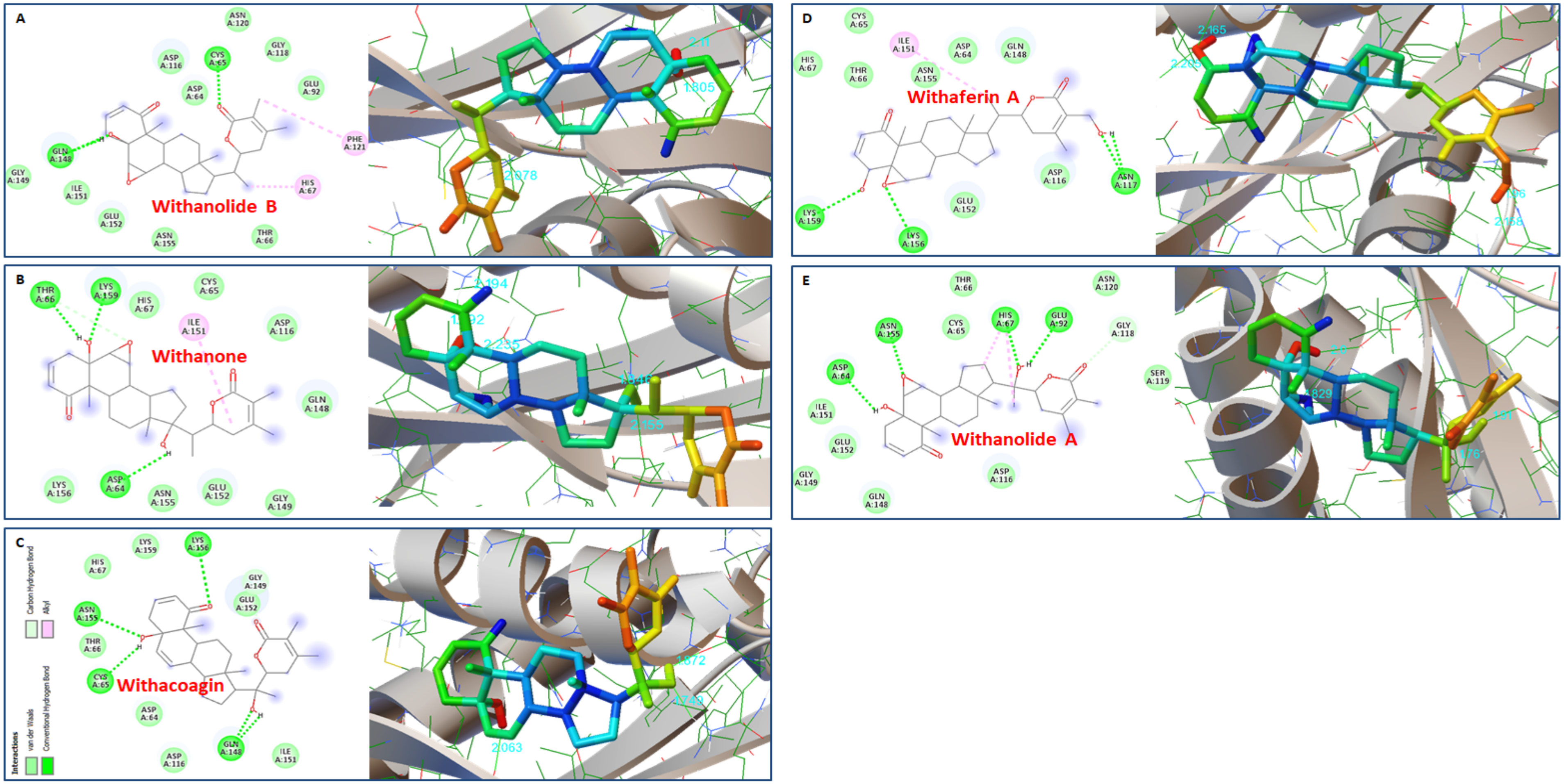

Figure S1. Molecular docking simulation analysis of HIV Integrase (PDB: 1QS4), with natural phytomolecules in *Withania somnifera*, in the active binding site. 2D interaction plot (left panel) and 3D map (right panel) of (**A**) Withanolide B (CID: 14236711), (**B**) Withanone (CID: 21679027), (**C**) Withacoagin (CID: 14236709), (**D**) Withaferin A (CID: 265237), and (**E**) Withanolide A (CID: 11294368).

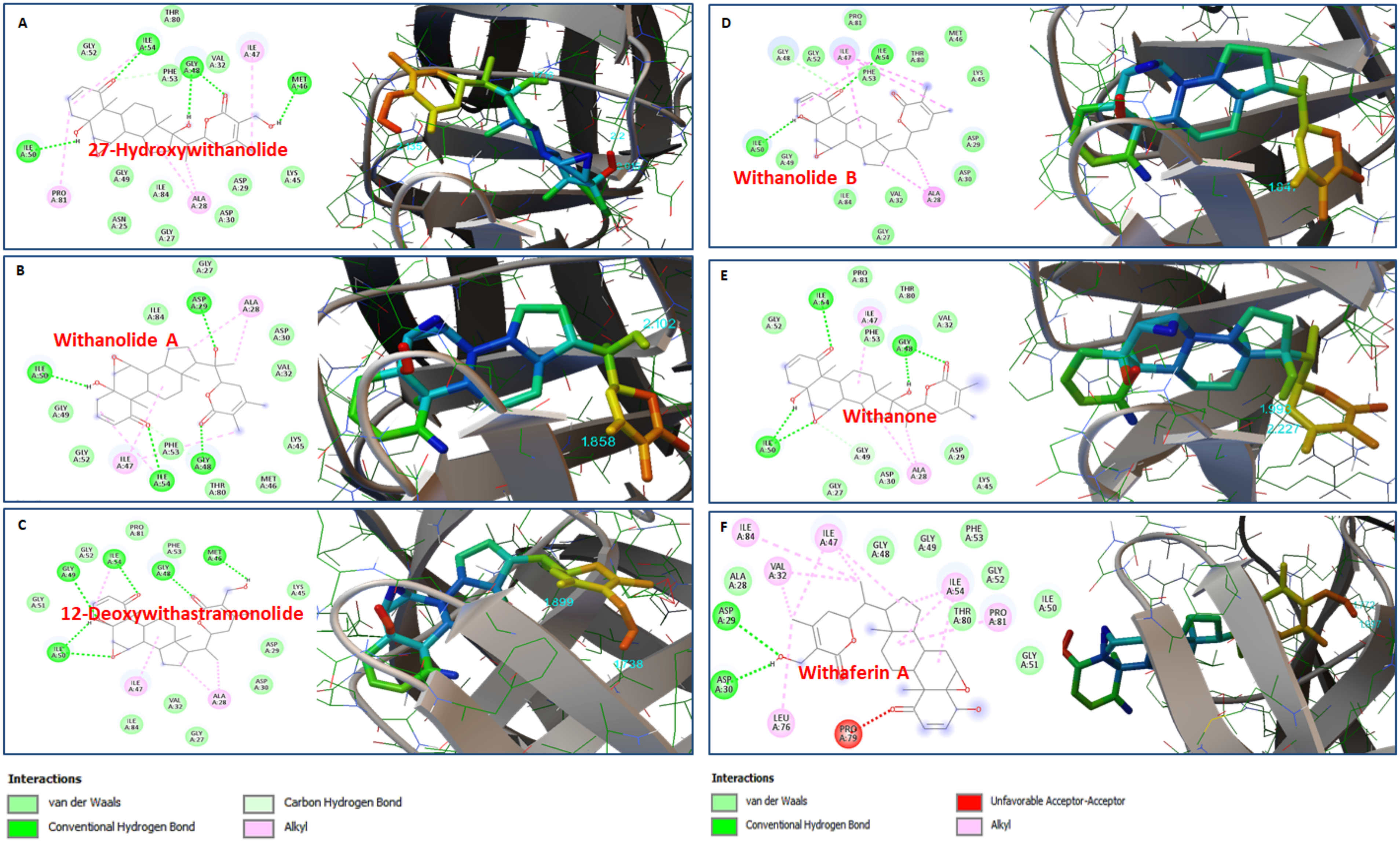

Figure S2. Molecular docking simulation analysis of HIV Protease (PDB: 5KR0), with natural phytomolecules in *Withania somnifera*, in the active binding site. Left panel (2D interaction plot) and right panel (3D map) (**A**) 27-Hydroxywithanone (CID: 21574483), (**B**) Withanolide A (CID: 11294368), (**C**) 12-Deoxywithastramonolide (CID: 44576309), (**D**) Withanolide B (CID: 14236711), (**E**) Withanone (CID: 21679027), and (**F**) Withaferin A (CID: 265237).

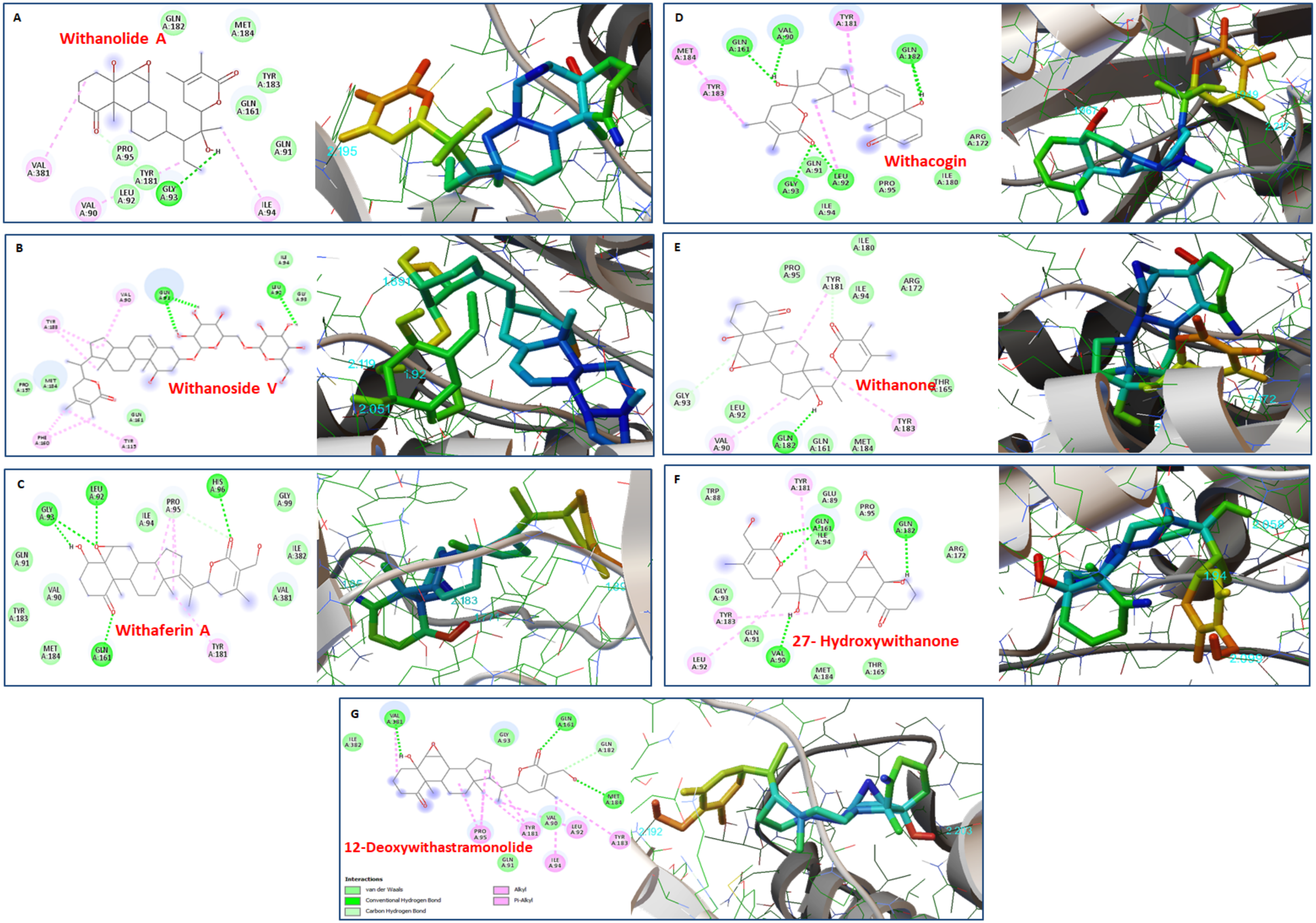

Figure S3. Molecular docking simulation analysis of HIV-1 Reverse Transcriptase (PDB: 3QIP) with natural phytomolecules from *Withania somnifera* and FDA approved drug Zidovudine in the active binding site. Left panel (2D interaction plot) and right panel (3D map) (**A**) Withanolide A (CID: 11294368), (**B**) Withanoside V (CID: 10700345), (**C**) Withaferin A (CID: 265237), (**D**) Withacoagin (CID: 14236709), (**E**) Withanone (CID: 21679027), (**F**) 27-Hydroxywithanone (CID: 21574483), and (**G**) 12-Deoxywithastramonolide (CID: 44576309).
